# Financial Incentives to Increase Colorectal Cancer Screening Uptake and Decrease Disparities: A Randomized Clinical Trial

**DOI:** 10.1001/jamanetworkopen.2019.6570

**Published:** 2019-07-03

**Authors:** Beverly B. Green, Melissa L. Anderson, Andrea J. Cook, Jessica Chubak, Sharon Fuller, Kilian J. Kimbel, Jeffrey T. Kullgren, Richard T. Meenan, Sally W. Vernon

**Affiliations:** Kaiser Permanente Washington, Seattle (Green); Kaiser Permanente Washington Health Research Institute, Seattle (Green, Anderson, Cook, Chubak, Fuller, Kimbel); Department of Family Medicine, University of Washington School of Medicine, Seattle (Green, Chubak); Department of Biostatistics, University of Washington School of Public Health, Seattle (Cook); Veterans Affairs Ann Arbor Healthcare System, Ann Arbor, Michigan (Kullgren); Department of Internal Medicine, University of Michigan Medical School, Ann Arbor (Kullgren); Center for Health Research, Kaiser Permanente Northwest, Portland, Oregon (Meenan); Department of Health Promotion and Behavior Sciences, University of Texas School of Public Health, Houston (Vernon).

## Abstract

**IMPORTANCE:**

Colorectal cancer screening rates are suboptimal, particularly among sociodemographically disadvantaged groups.

**OBJECTIVE:**

To examine whether guaranteed money or probabilistic lottery financial incentives conditional on completion of colorectal cancer screening increase screening uptake, particularly among groups with lower screening rates.

**DESIGN, SETTING, AND PARTICIPANTS:**

This parallel, 3-arm randomized clinical trial was conducted from March 13, 2017, through April 12, 2018, at 21 medical centers in an integrated health care system in western Washington. A total of 838 age-eligible patients overdue for colorectal cancer screening who completed a questionnaire that confirmed eligibility and included sociodemographic and psychosocial questions were enrolled.

**INTERVENTIONS:**

Interventions were (1) mail only (n = 284; up to 3 mailings that included information on the importance of colorectal cancer screening and screening test choices, a fecal immunochemical test [FIT], and a reminder letter if necessary), (2) mail and monetary (n = 270; mailings plus guaranteed $10 on screening completion), or (3) mail and lottery (n = 284; mailings plus a 1 in 10 chance of receiving $50 on screening completion).

**MAIN OUTCOMES AND MEASURES:**

The primary outcome was completion of any colorectal cancer screening within 6 months of randomization. Secondary outcomes were FIT or colonoscopy completion within 6 months of randomization. Intervention effects were compared across sociodemographic subgroups and self-reported psychosocial measures.

**RESULTS:**

A total of 838 participants (mean [SD] age, 59.7 [7.2] years; 546 [65.2%] female; 433 [52.2%] white race and 101 [12.1%] Hispanic ethnicity) were included in the study. Completion of any colorectal screening was not significantly higher for the mail and monetary group (207 of 270 [76.7%]) or the mail and lottery group (212 of 284 [74.6%]) than for the mail only group (203 of 284 [71.5%]) (*P* = .11). For FIT completion, interventions had a statistically significant effect (*P* = .04), with a net increase of 7.7% (95% CI, 0.3%–15.1%) in the mail and monetary group and 7.1% (95% CI, −0.2% to 14.3%) in the mail and lottery group compared with the mail only group. For patients with Medicaid insurance, the net increase compared with mail only in FIT completion for the mail and monetary or the mail and lottery group was 37.7% (95% CI, 11.0%–64.3%) (34.2% for the mail and monetary group and 40.4% for the mail and lottery group) compared with a net increase of only 5.6% (95% CI, −0.9% to 12.2%) among those not Medicaid insured (test for interaction *P* = .03).

**CONCLUSIONS AND RELEVANCE:**

Financial incentives increased FIT uptake but not overall colorectal cancer screening. Financial incentives may decrease screening disparities among some sociodemographically disadvantaged groups.

**TRIAL REGISTRATION:**

ClinicalTrials.gov identifier:

## Introduction

Colorectal cancer (CRC) is the second-leading cause of cancer-related death in the United States. Although CRC screening rates are steadily increasing, only 62% of age-eligible US adults are up to date for screening.^1^ Rates are lower among low-income (47%), Medicaid-insured (43%), uninsured (25%), African American (59%), Asian (52%), Native American (48%), and Hispanic (47%) populations.^1,2^ These rates fall short of the targets of 70% for Healthy People 2020^3^ and 80% for the National Colorectal Cancer Round Table.^4^

A systematic review and meta-analysis^5^ produced evidence from multiple studies and systematic reviews that indicated that patient-directed outreach and navigation interventions is associated with increased CRC screening rates. Multicomponent interventions are also effective, particularly when fecal tests are provided.^6^ Evidence is mixed on whether adding a financial incentive (such as cash) conditional on testing completion increases uptake, particularly among groups with lower screening rates (people of color and low-income groups).^7–11^

A previous study^12^ demonstrated that a low-cost, technology-facilitated program of mailed fecal tests and stepped-intensity support led to a 30% net increase in fecal testing in year 1. However, disparities persisted, particularly in nonwhite racial/ethnic groups and those not previously completing screening. We interviewed patients who received at least 3 mailed fecal tests during 3 years but completed none or only 1 test. Defensive information processing^13^ (eg, avoidance, procrastination) was a prominent reason for noncompletion.^14^ Thus, we hypothesized that offering incentives would lead to additional uptake, particularly among individuals who intended to screen but had not followed through. We conducted a 3-arm randomized clinical trial that compared mailed educational materials and fecal immunochemical tests (FITs) with mailed materials plus offers of 2 types of monetary incentives conditional on completion of CRC screening.

## Methods

The study was conducted and data collected from March 13, 2017, through April 12, 2018.^15^ Study procedures and protocol were approved by the Kaiser Permanente Washington (KPWA) Institutional Review Board. The study received a waiver of consent to identify, collect aggregate level data, and mail a survey to potentially eligible subjects. The trial protocol can be found in Supplement 1. This trial followed Consolidated Standards of Reporting Trials (CONSORT) reporting guideline (Figure).^16^

### Participants

The study took place at KPWA, an integrated health care system that provides health insurance and care to more than 700 000 individuals in Washington. Patients aged 50 to 75 years were potentially eligible if, based on claims and electronic health record (EHR) data, they were continuously enrolled in KPWA for at least 1 year; received care at 1 of 21 KPWA-owned medical centers in western Washington; were due for CRC screening with no history of CRC, colectomy, or inflammatory bowel disease; had no diagnosis of end-stage renal disease or dementia in the prior year; and had not been in a skilled nursing facility or hospice program in the prior year. *Due for CRC screening* was defined as no colonoscopy within 9 years and no fecal test within 12 months. A study sample of 10 000 patients was randomly selected from the pool of approximately 34 000 age-eligible individuals due for CRC screening, oversampling patients who were Medicaid insured or of nonwhite race or Hispanic ethnicity based on EHR data.

An introduction letter, an information sheet written in plain-language English that explained the study, and a baseline questionnaire were mailed to potentially eligible participants. The mailing included a $2 preincentive to encourage participation^17^ and a postage-paid return envelope. The survey included questions to confirm eligibility and collect demographic and psychosocial information. The questionnaire included the statement, “By returning this survey, I agree to participate in the Smart Options for Screening (SOS) program as described in the Information Sheet.”

We enrolled all patients who returned a completed questionnaire in English, regardless of their native language, and who did not self-report colonoscopy in the prior 9 years, prior CRC diagnosis, or first-degree blood relative with CRC diagnosed before 60 years of age. The first round of 5000 mailings yielded an 11.7% return rate (586 of 5000), which was lower than expected. A second round included a single-reminder telephone call to return the questionnaire (leaving a message when possible for persons not reached) for a 14.2% response rate (709 of 5000) (Figure).

### Randomization

After baseline surveys confirmed eligibility, participants were randomized to 1 of 3 study arms, stratified by clinic, self-reported prior CRC testing (yes or no), and nonwhite or Hispanic race/ethnicity (yes or no). The study programmer used a computer program to generate random allocation sequences, with the sequence concealed, using a block size of 6 within randomization strata. Study investigators were blinded to randomization arm until all 6-month outcome data were collected.

### Interventions

All participants received usual care, which at KPWA includes birthday reminder letters of needed screening (including CRC), receiving FIT kits at clinical visits, colonoscopy screening, and sometimes mailing FIT kits.^18^ Practitioners rarely order sigmoidoscopy, stool DNA, or virtual colonoscopy.

#### Arm 1: Mail Only

Mail only participants received up to 3 mailings. The initial mailing was an introductory letter and pamphlet with information on CRC screening importance, the benefits of finding CRC early and finding precancerous polyps and removing them before they become cancerous, and the advantages and disadvantages of CRC screening choices (FIT, flexible sigmoidoscopy, and colonoscopy). The letter indicated that recipients would soon receive a free FIT kit in the mail and included a toll-free number to call if they did not want a kit or wanted more information. The second mailing, sent 1 week later, was the FIT kit (1-sample OC-Auto FIT; Polymedco), wordless pictographic instructions,^19^ a postage-paid return envelope, and a letter stating that they should contact their physician’s office if they preferred a different screening option (eg, colonoscopy). Individuals not completing FIT within 3 weeks received a reminder letter.

#### Arm 2: Mail and Monetary

Arm 2 participants received the same interventions as arm 1 (mail only) but were told in each mailing that they would receive $10 cash (guaranteed incentive) if they completed FIT or another CRC screening test.

#### Arm 3: Mail and Lottery

Arm 3 participants received the same interventions as arm 1 (mail only) but were told in each mailing that they would be entered into a lottery with a 1 in 10 chance of winning $50 cash (probabilistic incentive) if they completed FIT or another CRC screening test.

Both arm 2 (mail and monetary) and arm 3 (mail and lottery) reminder letters informed participants that they needed to complete screening within 5 months to be eligible for the financial incentive.

### Outcome Measures

The CRC screening completion was determined using automated data (procedure codes, laboratory results, and claims data) from the time of sample pull until 6 months after randomization. Administrative and EHR data were used to capture age, sex, race/ethnicity, insurance type, Charlson Comorbidity Index score,^20^ and body mass index. The eligibility and baseline questionnaire collected information on race/ethnicity, educational level, income, marital status, employment status, tobacco use,^21^ and prior completion of CRC testing.^22^ Health literacy was measured using a validated single-item question.^23^ In analyses, race was defined based on automated data, which was more complete (race missing for 14 participants) than self-reported data, which was used if race was missing from automated data.

The baseline questionnaire (eTables 1–3 in Supplement 2) assessed psychosocial constructs, including perceived risk of CRC, advantages and disadvantages of completing different CRC tests, self-efficacy for completing CRC screening (using a question validated in diverse settings),^24–29^ and defensive information processing opt-out behavior (eg, “If I feel healthy, I do not go to the doctor for a routine check-up”).^13^ We also assessed dispositional optimism (eg, “In uncertain times I usually expect the best” vs “If something can go wrong for me, it will”), which is associated with health maintenance behaviors.^30^ Two items were derived from the 14-item Consideration of Future Consequences scale, which assesses distant vs immediate consequences of actions (“I make decisions based on how easy they are to do” and “I only act to take care of immediate concerns, figuring the future will take care of itself”).^31^

### Outcomes

The primary outcome was completion of any CRC testing (FIT, flexible sigmoidoscopy, or colonoscopy) within 6 months of randomization (yes or no). The prespecified secondary outcomes were completion of FIT or colonoscopy testing within 6 months. If participants completed more than 1 test (eg, FIT and colonoscopy), the first test completed was counted.

### Statistical Analysis

The initial target sample size of 1150 participants was designed to provide 80% power to detect a 10% difference in screening rates among randomized groups. A lower-than-expected response rate to the eligibility survey resulted in a sample of 898 enrolled participants. The final analytic sample of 838 provided 80% power to detect a 11.2% difference between groups, assuming a screening rate of 60% in the mail only group.

Analyses followed a modified intention-to-treat approach, with all study participants included in analyses except those randomized in error (Figure). Generalized linear models with logit link and robust SEs were used to estimate intervention effects on screening rates. We used logistic regression model results to estimate mean marginal effects, reported as differences in adjusted estimated screening rates between randomized groups. Models were adjusted for prespecified participant characteristics, including age, sex, race, and self-report of prior CRC test completion. We conducted sensitivity analyses in which we excluded randomized participants who completed testing after initial mailing of the eligibility questionnaire but before randomization (n = 92) (Figure).

Secondary post hoc analyses explored whether effects of financial incentives on screening uptake differed by participant characteristics. We prespecified race, Medicaid insurance, ever completion of previous CRC screening, educational level, income, and behavioral constructs, including self-efficacy for screening completion and defensive information processing as important potential effect modifiers based on our prior research.^12^ Interaction terms between these variables and intervention groups were included in multivariable models to estimate intervention effects within subgroups. Wald tests were used to assess overall significance of effect modification. A 2-sided test with *P* < .05 was considered to be statistically significant. Analyses used Stata statistical software, version 15.0 (StataCorp).

## Results

### Recruitment and Enrollment

We mailed 10 000 letters to individuals aged 50 to 75 years who, based on automated data, were not current for CRC screening; 1295 returned questionnaires (Figure). Of these 1295 individuals, 397 (30.7%) were ineligible (395 self-reported colonoscopy within 9 years, and 2 reported a prior CRC diagnosis); 898 were enrolled and randomized to (1) mail only, (2) mail and monetary, or (3) mail and lottery. Subsequently, 60 individuals randomized in error were excluded from analyses: 5 reported completion of colonoscopy in the prior 9 years (found after re-review of baseline surveys), 14 reported a first-degree relative with CRC before 60 years of age, and 41 completed CRC screening after sample selection but before the initial study mailing (before the invitation letter and eligibility survey). The CRC screening completion status was not known at randomization because of lags in obtaining claims and EHR data. Individuals randomized in error were distributed similarly across arms. The primary analysis (n = 838; mean [SD] age, 59.7 [7.2] years; 546 [65.2%] female, 433 [52.2%] white race and 101 [12.1%] Hispanic ethnicity) included 284 randomized to mail only, 270 randomized to the mail and monetary intervention, and 284 randomized to the mail and lottery intervention. The primary analysis included 92 randomized individuals who completed CRC screening after the initial study mailing but before receiving any intervention mailings; sensitivity analyses estimated intervention effects excluding this group, resulting in 248 randomized to mail only, 239 randomized to the mail and monetary intervention, and 259 randomized to the mail and lottery intervention. Baseline characteristics were similar among randomization arms (Table 1).

### Primary and Secondary Outcomes

The number of individuals completing any CRC screening within 6 months (primary outcome), was high among all 3 groups and not significantly higher for the mail and monetary (207 of 270 [76.7%]) and mail and lottery (212 of 284 [74.6%]) incentives groups than the mail only group (203 of 284 [71.5%]) (*P* = .11) (Table 2). The intervention effect for the secondary outcome of FIT completion was statistically significant (*P* = .04), with an adjusted net increase of 7.7% (95% CI. 0.3%–15.1%) for the mail and monetary group and 7.1% (95% CI, −0.2% to 14.3%) for the mail and lottery group compared with the mail only group. Conversely, colonoscopy completion rates were higher among the mail only group (15 of 284 [5.3%]) compared with the mail and monetary group (9 of 270 [3.3%]) and the mail and lottery group (8 of 284 [2.8%]), but this difference was not significant (*P* = .23).

### Sensitivity Analysis

We performed sensitivity analyses removing 92 participants who completed CRC testing ordered by their physician after study invitation but before randomization. The results were similar to the those of the primary analysis. The net increase in any CRC test completion was not significant: 7.0% (95% CI, −0.9% to 14.8%) in the mail and monetary group and 6.2% (95% CI, −1.4% to 13.8%) in the mail and lottery group compared with the mail only group. There was a statistically significant net increase in FIT completion of 8.6% (95% CI, 0.7%–16.6%) in the mail and lottery group and 8.5% (95% CI, 0.4%–16.6%) in the mail and monetary group compared with the mail only group (eTable 1 in the Supplement).

### Subgroup Analyses

We estimated the effects of the mail and monetary and mail and lottery interventions separately in the prespecified subgroup analyses (eTable 2 and eTable 3 in the Supplement). We selected FIT completion as the outcome variable for subgroup analyses because financial incentives did not significantly affect colonoscopy completion rates. Because the effects of these incentives were generally similar, we reported the combined effect of any financial incentive (mail and monetary or mail and lottery) compared with mail only in Table 3 and Table 4.

Patients with Medicaid were significantly more responsive to financial incentives, with a net increase of 37.7% (95% CI, 11.0%–64.3%) for mail and monetary and mail and lottery combined compared with a 5.6% (95% CI, −0.9% to 12.2%) increase among those with other insurance (interaction effect *P* = .03) (Table 3). Individuals who had previously completed CRC screening had higher FIT completion rates than those who had never been screened, but the intervention effect did not differ significantly (difference for mail and incentive vs mail only, 8.4 [95% CI, 1.4–15.4] for those with screening vs 3.8 [95% CI, −9.4 to 16.9] for those without; *P* = .34).

Financial incentives led to significant increases in FIT uptake among individuals with low dispositional optimism or high opt-out defensive processing, along with those who reported making decisions based on their ease or reported their risk of CRC was much lower than others their age (Table 4). However, differences in intervention effects between subgroups were significant only for a single item from the 14-item Consideration of Future Consequences Scale (“I make decisions or take actions based on how easy they are to do”). We did not see differences in intervention effects by self-reported barriers to, benefits of, or self-efficacy for completing CRC testing.

## Discussion

Our study enrolled an ethnically diverse cohort of patients who were overdue for CRC screening to test whether 2 types of monetary incentives increased CRC screening uptake compared with mailed FITs only. The interventions did not increase overall CRC screening uptake by 6 months; however, the increase in FIT completion was statistically significant.

Patients may have chosen FIT over colonoscopy because it was an easier, faster way to get the incentive or because it was an easier test to complete. All patients were fully insured with no out-of-pocket costs for FIT but might have incurred some costs for screening or diagnostic colonoscopy (after a positive FIT result), depending on their coverage.^32^

Even among patients with no out-of-pocket costs for colonoscopy, the incentives may have been insufficient to encourage colonoscopy completion. Mehta et al^7^ randomized 2250 employees in a work-based insurance plan with colonoscopy fully covered to receive an email with a web link or a telephone number for scheduling a colonoscopy or the same email plus an offer of $100, conditional on colonoscopy completion. The $100 incentive led to a small, significant increase in colonoscopy at 3 months compared with email only (3.7% vs 1.6%), suggesting a modest intervention benefit. Slater et al^10^ randomized 94 294 Minnesota Medicaid patients overdue for CRC screening to receive information, a telephone number to a navigator (who addressed colonoscopy barriers and scheduled colonoscopies), and a $20 incentive for completing colonoscopy vs delayed intervention. The increase in colonoscopy completion was small but significant (absolute difference, 0.3%). An upstate New York Medicaid Managed Care program randomized 7123 patients to receive mailed reminders to complete CRC screening, reminders plus $25 gift card for completing any type of CRC test, or no reminders. No significant differences occurred among the groups, possibly because no FITs were provided and the incentive might have not been large enough to provide the added motivation needed to complete colonoscopy.^33^ Our study found a nonsignificant trend toward fewer colonoscopies. Possible reasons are that individuals with sufficient intrinsic motivation to complete screening colonoscopy did not need incentives, some individuals were discouraged by possible additional charges, or some chose FIT when it was directly mailed to them.

Three prior trials focused on using financial incentives to increase FIT uptake. At 2 Veterans Affairs primary care clinics, the distribution of fecal tests at visits were assigned based on day of the week to an arm that included a card that informed the patient of an incentive ($5, $10, 1 in 10 chance of $50, or entry into a $500 raffle) or to a control, no-incentive arm.^8^ Only the 1 in 10 chance of $50 led to a significant increase in 30-day test completion (19.6% increase compared with controls, with no significant changes for other incentives). Screening uptake overall was lower than in our study (range, 29.7%–49.3%), possibly because the FIT used required 3 days of testing (rather than the single sample used in our study), and patients were followed up for only 30 days to determine test completion. No interaction effects were seen between previous FIT adherence and incentives. In a safety-net health care system, patients overdue for CRC screening were randomized to receive a mailed single-sample FIT or this plus either a $5 or $10 Walmart gift card.^9^ One-year FIT completion rates were not significantly different among the groups: 34.6% in the mail-only group and 36.2% and 39.2% for increasing gift-card amounts. The FIT uptake was lower overall compared with our study, with no subgroup differences by sex, age, ethnicity, or neighborhood poverty rates. An urban, academic primary care population of primarily low-income and nonwhite patients overdue for CRC screening was randomized to receive mailed FIT only or mailed FIT and an incentive: $10 gift card (unconditional), $10 gift card conditional on FIT completion, or entry into a raffle with a 1 in 10 chance of winning $100 conditional on FIT completion.^11^ The FIT completion rates were not significantly different among the groups (26% for the mail only group, 27.2% for the unconditional incentive group, 23.2% for the conditional incentive group, and 17.0% for the conditional lottery group) or by sex or race.

Discordant results among these studies and ours might be explained by the association between behavioral incentive constructs and context. Direct mailing of FIT kits to patients is a nudge; it makes desired activities easier to do. Adding financial incentives offsets present time–biased preferences, which is the natural tendency to devalue gains received later. For individuals who were somewhat inclined to complete CRC screening but had not prioritized it, the incentive may have tipped the balance toward completion, at least for the simpler FIT. Context might also be important. The CRC screening rates are high at KPWA. Standard practice at all primary care visits includes giving patient FIT kits if they are overdue for CRC screening. Many people in our study completed FITs that their practitioner ordered and they had at home before they received any interventions or study FITs. Participants may already have had some intrinsic motivation to complete screening (eg, practitioner recommendation). Inviting individuals to the study served as a reminder. Individuals with low awareness or less experience with CRC screening or little intrinsic motivation might not respond to nudges with or without incentives. Our results suggest that colonoscopy completion might require greater intrinsic motivation, larger incentives,^7^ or additional interventions.^10^

### Strengths and Limitations

Collection of patient-reported psychosocial data was a strength. We found that participants who made decisions based on the ease of actions were more responsive to financial incentives. This finding suggests that the incentives worked best as extrinsic motivators when they aligned with the intrinsic motivations of participants who were not opposed to convenient FIT screening but did not prioritize it. More intensive interventions might be needed in other settings (safety-net clinics, urban inner-city clinics) and for populations with less intrinsic motivation (never screened). In addition, it is unknown whether financial incentives would need to be continued over time to ensure long-term adherence to FIT screening.

A limitation of our study was that patients were required to return a baseline questionnaire to be eligible. Return rates were lower than anticipated, with modest improvements after adding a reminder call. Thus, our results may not be generalizable to individuals nonresponsive to mailed surveys and reminder calls. Patients received $2 in the invitation letter; thus, those returning the baseline questionnaire might have been more responsive to financial incentives. In addition, CRC screening rates are high in our health care system, and patients overdue for screening may be more responsive than in other settings where baseline screening rates are lower. The subgroup analyses demonstrated that Medicaid-insured individuals were significantly more responsive to incentives than non-Medicaid participants. Point estimates also suggest that Hispanic and nonwhite groups may be more responsive to incentives than non-Hispanic and white groups, but differences were not significant. In the mail only group, FIT uptake was less than 43% among Medicaid insured, with incentives leading to FIT uptake higher than 82%. Although reductions in screening disparities are encouraging, the secondary analyses were hypothesis generating and included multiple comparisons that could lead to spurious significance. Also, this study may have been underpowered to find a significant difference in the primary outcome, colonoscopy or FIT completion combined, and to find significant differences in intervention effects among subgroups.

## Conclusions

Adding guaranteed or probabilistic financial incentives led to increased FIT completion rates but not CRC screening overall in a mailed CRC screening program within an integrated health care system. Incentive effects were greater among Medicaid-insured patients. Larger studies are needed to confirm our findings in other settings.

## Supplementary Material

Supplement 1 Trial Protocol

Supplement 2 **eTable 1.** Proportions Tested for Colorectal Cancer Within 6 Months From Sensitivity Analysis Excluding Participants Who Tested After Questionnaires Were Mailed but Before Randomization and Receiving Information About Incentives
**eTable 2.** Subgroup Analyses of Intervention Effects on FIT Completion by Sociodemographic Characteristics With Separate Estimates of the Effects of Mail and Monetary and Mail and Lottery Interventions
**eTable 3.** Subgroup Analyses of Intervention Effects on FIT Completion by Psychosocial Measures Self-Reported at Baseline With Separate Estimates of the Effects of Mail and Monetary and Mail and Lottery Interventions

Supplement 3 Data Sharing Statement

Supplementary Online Content

## Figures and Tables

**Figure.**
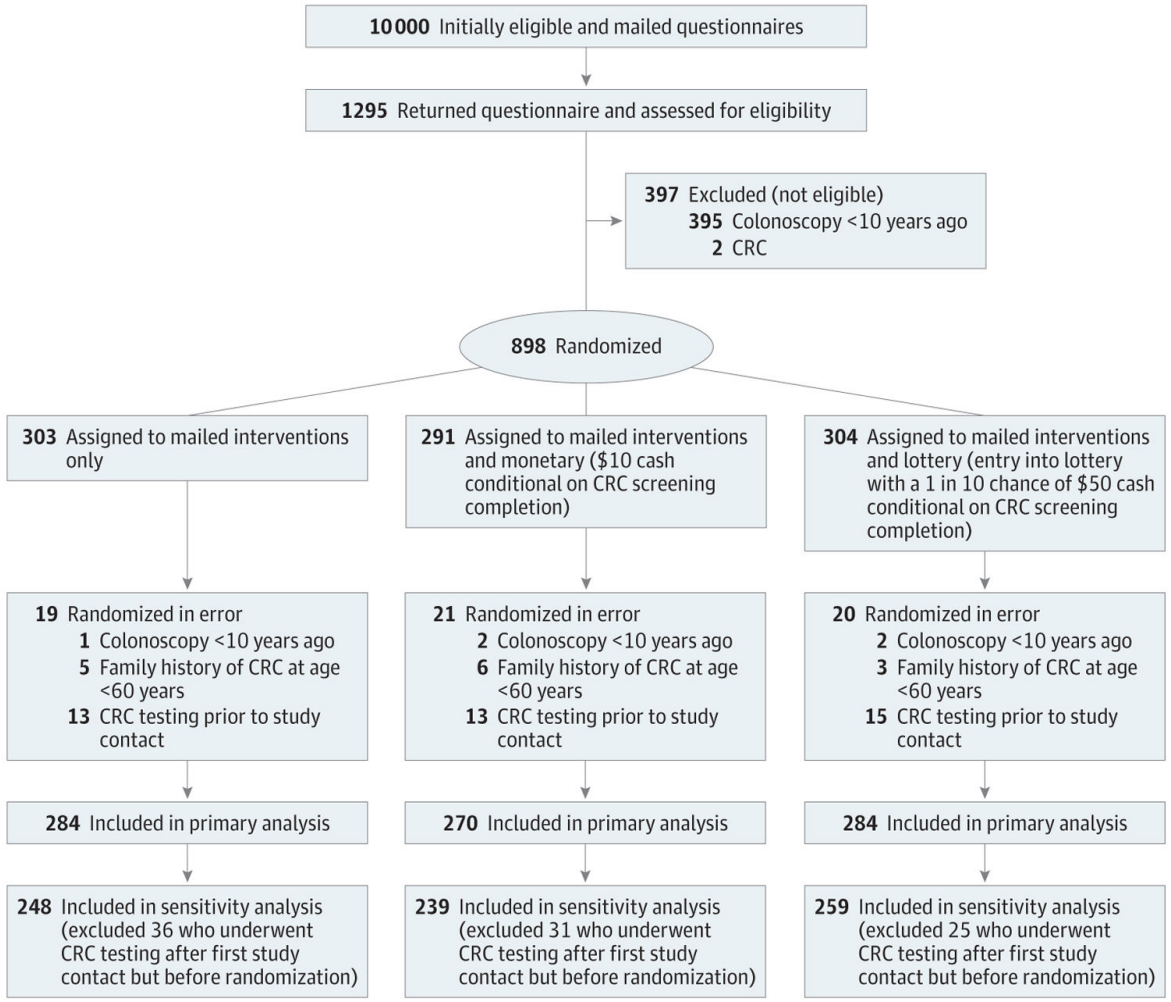
CONSORT Study Flow Diagram CRC indicates colorectal cancer.

**Table 1.** Baseline Characteristics of Study Participants^a^

	No. (%) of Participants			
Characteristic	Mail Only (n = 284)	Mail and Monetary (n = 270)^b^	Mail and Lottery (n = 284)^c^	Total (N = 838)
Age ≥60 y	137 (48.2)	123 (45.6)	143 (50.4)	403 (48.1)
Female	177 (62.3)	180 (66.7)	189 (66.5)	546 (65.2)
Race^d^				
White	139 (49.1)	146 (54.7)	148 (52.9)	433 (52.2)
Black	46 (16.3)	41 (15.4)	37 (13.2)	124 (14.9)
Asian	76 (26.9)	59 (22.1)	73 (26.1)	208 (25.1)
Other	22 (7.8)	21 (7.9)	22 (7.9)	65 (7.8)
Hispanic ethnicity	28 (9.9)	40 (14.9)	33 (11.7)	101 (12.1)
Literacy^e^				
Never, rarely	246 (87.9)	233 (87.9)	232 (84.1)	711 (86.6)
Sometimes, often, always	34 (12.1)	32 (12.1)	44 (15.9)	110 (13.4)
Medicaid insured	14 (4.9)	22 (8.1)	23 (8.1)	59 (7.0)
Annual household income				
≤$50 000	99 (37.6)	91 (36.0)	102 (40.0)	292 (37.9)
>$50 000	164 (62.4)	162 (64.0)	153 (60.0)	479 (62.1)
Educational level				
High school or less	41 (14.9)	37 (14.1)	57 (20.8)	135 (16.6)
Some college	90 (32.6)	90 (34.4)	91 (33.2)	271 (33.4)
College degree or higher	145 (52.5)	135 (51.5)	126 (46.0)	406 (50.0)
Prior CRC screening^f^	208 (73.2)	196 (72.6)	206 (72.5)	610 (72.8)
Current tobacco user	20 (7.1)	20 (7.7)	28 (10.0)	68 (8.3)
BMI				
<25	80 (28.8)	85 (32.3)	72 (25.8)	237 (28.9)
25 to <35	151 (54.3)	136 (51.7)	158 (56.6)	445 (54.3)
≥35	47 (16.9)	42 (16.0)	49 (17.6)	138 (16.8)
Charlson Comorbidity Index score^g^				
0	190 (74.8)	180 (74.1)	178 (70.1)	548 (73.0)
1	40 (15.7)	34 (13.2)	45 (17.7)	117 (15.6)
≥2	24 (9.4)	31 (12.8)	31 (12.2)	86 (11.5)
Risk of CRC in the next 10 y compared with other same-aged people				
Much lower	93 (33.8)	84 (32.2)	100 (36.8)	277 (34.3)
A little lower	75 (27.3)	76 (29.1)	67 (24.6)	218 (27.0)
Average	90 (32.7)	89 (34.1)	91 (33.5)	270 (33.4)
A little or much higher	17 (6.2)	12 (4.6)	14 (5.1)	43 (5.3)
No. of primary care visits in 2017				
0	75 (26.4)	74 (27.4)	81 (28.5)	230 (27.4)
1	89 (31.3)	73 (27.0)	80 (28.2)	242 (28.9)
2	59 (20.8)	55 (20.4)	52 (18.3)	166 (19.8)
≥3	61 (21.5)	68 (25.2)	71 (25.0)	200 (23.9)

Abbreviations: BMI, body mass index (calculated as weight in kilograms divided by height in meters squared); CRC, colorectal cancer.

a Missing values were excluded when computing column percentages: race (n = 8), Hispanic ethnicity (n = 6), literacy (n = 17), income (n = 67), educational level (n = 26), tobacco (n = 18), BMI (n = 18), Charlson Comorbidity Index score (n = 87), employment (n = 23), marital status (n = 21), and risk of CRC (n = 30).

b Mailed interventions plus $10 cash incentive conditional on completion of CRC screening.

c Mailed interventions plus entry into a lottery with a 1 in 10 chance of winning $50 conditional on completion of colorectal cancer screening.

d American Indian, Pacific Islander, multiracial, or other.

e “How often do you need to have someone help when you read instructions, pamphlets, or other written materials from your doctor or pharmacy?”

f Prior CRC screening defined as a self-report of prior completion of fecal testing, colonoscopy, or flexible sigmoidoscopy.

g Scores ranged from 0 to 8 among study participants, with higher scores indicating greater comorbidities.

**Table 2.** Proportions of Participants Completing CRC Screening Tests Within 6 Months

	No. (%) of Participants			Adjusted for Age, Sex, Race, and Prior Screening		
CRC Test	Mail Only (n = 284)	Mail and Monetary (n = 270)^a^	Mail and Lottery (n = 284)^b^	Difference for Mail and Monetary vs Mail Only (95% CI)	Difference for Mail and Lottery vs Mail Only (95% CI)	Global *P* Value
Any CRC test^c^	203 (71.5)	207 (76.7)	212 (74.6)	5.7 (−1.3 to 12.8)	4.4 (−2.5 to 11.3)	.11
FIT^d^	188 (66.2)	198 (73.3)	204 (71.8)	7.7 (0.3 to 15.1)	7.1 (−0.2 to 14.3)	.04
Colonoscopy^e^	15 (5.3)	9 (3.3)	8 (2.8)	−2.1 (−5.5 to 1.3)	−2.7 (−6.1 to 0.6)	.23

Abbreviations: CRC, colorectal cancer; FIT, fecal immunochemical test.

a Mailed interventions plus $10 cash incentive conditional on completion of CRC screening.

b Mailed interventions plus entry into a lottery with a 1 in 10 chance of winning $50 conditional on completion of CRC screening.

c Colonoscopy or FIT (no participants had flexible sigmoidoscopies).

d Fecal immunochemical test completion as the first test (does not include colonoscopy followed by an FIT).

e Colonoscopy completion as first test (does not include colonoscopy following an FIT).

**Table 3.** Subgroup Analyses of Intervention Effects on FIT Completion by Sociodemographic Characteristics

Characteristic	Screened Within 6 mo, No./Total No. (%)		Adjusted Differences Between Groups	
	Mail Only	Mail and Incentive^a^	Difference for Mail and Incentive vs Mail Only (95% CI)	*P* Value^b^
Sex				
Male	77/107 (72.0)	142/185 (76.8)	4.5 (−5.8 to 14.8)	.61
Female	111/177 (62.7)	260/369 (70.5)	9.0 (0.8 to 17.1)	
Age, y				
<60	100/147 (68.0)	209/288 (72.6)	4.6 (−3.8 to 13.0)	.59
≥60	88/137 (64.2)	193/266 (72.6)	9.1 (−0.3 to 18.5)	
Hispanic ethnicity				
No	170/254 (66.9)	347/477 (72.7)	7.1 (0.4 to 13.8)	.43
Yes	16/28 (57.1)	54/73 (74.0)	16.7 (−5.5 to 38.8)	
Race				
White	96/139 (69.1)	214/294 (72.8)	5.0 (−4.0 to 13.9)	.68
Black	28/46 (60.9)	58/78 (74.4)	14.8 (−1.7 to 31.3)	
Asian	48/76 (63.2)	95/132 (72.0)	10.5 (−2.3 to 23.3)	
Other	15/22 (68.2)	30/43 (69.8)	1.0 (−21.1 to 23.1)	
Literacy^c^				
Never, rarely	166/246 (67.5)	345/465 (74.2)	7.6 (0.7 to 14.5)	.81
Sometimes, often, or always	18/34 (52.9)	49/76 (64.5)	10.9 (−8.0 to 29.8)	
Medicaid				
No	182/270 (67.4)	365/509 (71.7)	5.6 (−0.9 to 12.2)	.03
Yes	6/14 (42.9)	37/45 (82.2)	37.7 (11.0 to 64.3)	
Annual household income				
<$50 000	58/99 (58.6)	137/193 (71.0)	13.2 (2.4 to 24.0)	.32
≥$50 000	113/164 (68.9)	234/315 (74.3)	5.6 (−2.9 to 14.1)	
Educational level				
Less than high school	27/41 (65.9)	67/94 (71.3)	6.2 (−8.4 to 20.7)	.16
Some college	50/90 (55.6)	131/181 (72.4)	16.7 (4.8 to 28.6)	
College degree or higher	105/145 (72.4)	192/261 (73.6)	1.7 (−7.3 to 10.7)	
Prior completion of colorectal cancer screening				
No	38/76 (50.0)	81/152 (53.3)	3.8 (−9.4 to 16.9)	.34
Yes	150/208 (72.1)	321/402 (79.9)	8.4 (1.4 to 15.4)	
BMI				
<25	52/80 (65.0)	121/157 (77.1)	13.1 (0.8 to 25.3)	.48
25 to <35	104/151 (68.9)	215/294 (73.1)	5.8 (−2.9 to 14.4)	
≥35	30/47 (63.8)	59/91 (64.8)	2.9 (−13.2 to 19.0)	
Current tobacco use				
No	174/260 (66.9)	362/492 (73.6)	8.1 (1.3 to 14.9)	.90
Yes	10/20 (50.0)	31/48 (64.6)	7.5 (−17.2 to 32.2)	
Charlson Comorbidity Index score^d^				
0	131/190 (68.9)	262/358 (73.2)	7.1 (−0.8 to 14.9)	
1	30/40 (75.0)	62/77 (80.5)	4.3 (−10.7 to 19.3)	.92
≥2	13/24 (54.2)	39/62 (62.9)	12.4 (−9.6 to 34.5)	

Abbreviations: BMI, body mass index (calculated as weight in kilograms divided by height in meters squared); FIT, fecal immunochemical test.

a Cash incentive ($10) or lottery incentive (1 in 10 chance of winning $50) conditional on FIT completion combined.

b *P* value for the difference in intervention effect across subgroups.

c “How often do you need to have someone help when you read instructions, pamphlets, or other written materials from your doctor or pharmacy?”

d Scores ranged from 0 to 8 among study participants, with higher scores indicating greater comorbidities.

**Table 4.** Subgroup Analyses of Intervention Effects on Fecal Immunochemical Test Completion by Psychosocial Measures Self-reported at Baseline

Psychosocial Measure	Screened Within 6 mo, No./Total No. (%)		Adjusted Differences Between Groups	
	Mail Only	Mail and Incentive^a^	Difference for Mail and Incentive vs Mail Only (95% CI)	*P* Value^b^
Barriers to CRC screening^c^				
1 to <2	93/137 (67.9)	173/231 (74.9)	9.3 (−0.2 to 18.8)	.94
2 to <3	62/92 (67.4)	152/202 (75.2)	7.8 (−3.0 to 18.7)	
3–5	28/50 (56.0)	66/102 (64.7)	6.6 (−9.0 to 22.1)	
Benefits of CRC screening^c^				
1 to <3	17/31 (54.8)	29/53 (54.7)	1.2 (−20.6 to 23.1)	.69
3 to <4	51/78 (65.4)	120/168 (71.4)	8.1 (−4.3 to 20.6)	
4–5	114/169 (67.5)	243/316 (76.9)	9.8 (1.6 to 18.0)	
Self-efficacy for completing CRC screening^c^				
1 to <3	16/33 (48.5)	31/59 (52.5)	7.4 (−12.4 to 27.1)	.57
3 to <4	57/80 (71.3)	103/141 (73.0)	2.9 (−8.8 to 14.7)	
4–5	109/165 (66.1)	257/335 (76.7)	11.0 (2.5 to 19.5)	
General dispositional optimism^d^				
Low	35/62 (56.5)	91/119 (76.5)	20.6 (6.0 to 35.1)	.08
Moderate	65/107 (60.7)	146/205 (71.2)	9.7 (−0.7 to 20.2)	
High	78/103 (75.7)	147/203 (72.4)	−0.3 (−10.9 to 10.3)	
Defensive information processing: opt-out behavior score^e^				
Lowest fertile	62/94 (66.0)	127/172 (73.8)	7.9 (−3.8 to 19.5)	.17
Middle fertile	83/110 (75.5)	154/202 (76.2)	1.7 (−8.0 to 11.4)	
Highest fertile	38/75 (50.7)	113/166 (68.1)	18.6 (5.6 to 31.5)	
Single item from the 14-item Consideration of Future Consequences scale: “1 make decisions or take actions based on how easy they are to do”				
Not at all or somewhat not	111/155 (71.6)	198/282 (70.2)	−2.0 (−10.9 to 6.8)	.003
Uncertain	24/40 (60.0)	62/86(72.1)	18.0 (0.6 to 35.5)	
Somewhat or very much	48/84 (57.1)	129/166 (77.7)	21.3 (10.1 to 32.5)	
Risk of CRC in the next 10 y compared with other same-aged people				
Much lower	58/93 (62.4)	134/184 (72.8)	12.5 (1.5 to 23.6)	.55
A little lower	53/75 (70.7)	109/143 (76.2)	8.1 (−4.2 to 20.5)	
Average	60/90 (66.7)	130/180 (72.2)	4.3 (−7.0 to 15.6)	
A little or much higher	10/17 (58.8)	14/26 (53.8)	6.5 (−35.8 to 22.9)	

Abbreviation: CRC, colorectal cancer.

a Cash incentive ($10) or lottery incentive (1 in 10 chance $50) conditional on FIT testing completion combined.

b *P* value for the difference in intervention effect across subgroups.

c Mean score with a range of 1 to 5 indicating low to high self-reported barriers.

d Score for low was 0 to 13; moderate, 14 to 18; and high, 19 to 24.

e Mean of 3 items with a range of 1 to 5, with 2 or higher indicating the lowest tertile; higher than 2 to 3.5, middle tertile; and higher than 3.5, highest tertile.

## Key Points

### Question

Do financial incentives increase uptake of colorectal cancer screening in patients overdue for colorectal cancer screening?

### Findings

In this 3-arm randomized clinical trial of 838 adults who were overdue for colorectal cancer screening, incentives did not significantly increase colorectal cancer screening but significantly increased fecal immunochemical test completion.

### Meaning

Financial incentives did not increase uptake of colorectal screening.
