# Supplementary material for: Financial Incentives to Increase Colorectal Cancer Screening Uptake and Decrease Disparities: A Randomized Clinical Trial
Source: JAMA Netw Open. Author manuscript; Available in PMC 2019 Oct 12. (PMC6789432; doi:10.1001/jamanetworkopen.2019.6570)
Supplement: Supplement 1 — Trial Protocol [file NIHMS1047899-supplement-Supplement_1.pdf]

1. Protocol Title

Financial Incentives to Increase Colorectal Cancer Screening in Priority Populations

2. Objectives

We propose to build on the success of the Systems of Support to Increase Colorectal Cancer trial (SOS, R01CA121125, Green) and focus on disparity groups who are less likely to be current for CRC screening. We will test whether financial incentives increase screening uptake and decrease screening disparities. Usual care for CRC screening at Kaiser Permanente Washington (KPWA, previously Group Health Cooperative) includes giving fecal immunochemical test (FITs) to patients at clinic visits and mailing FITs during patients' birthday month.

We will send letters and a questionnaire to 5000 patients aged 50-75 who are not current for CRC screening, inviting them to participate in a study to increase screening. Using race, ethnicity, and insurance coverage electronic health record (EHR) data, two-thirds of those invited will be identified as non-White race or Hispanic ethnicity or having Medicaid coverage. The other third will be white and have non-Medicaid insurance. coverage. Invitees who return the survey and remain eligible (estimated N = 1150) will be randomized to either (1) Mailed fecal immunochemical test (FIT) kit, or mailed FIT kit *plus* one of two types of financial incentives for completing testing: (2) a guaranteed Monetary incentive ($10), or (3) probabilistic Lottery incentive (1 in 10 chance for $50). Our primary outcome will be completion of CRC screening within 6 months. Secondary analyses will assess whether the effect of incentives on screening completion rates differs by race/ethnicity and Medicaid coverage.

3. Background

Strong evidence from multiple studies and systematic reviews supports that direct mailing fecal tests increases CRC screening rates.[1] We demonstrated that a low-cost, health information technology-facilitated trial of mailed fecal tests and stepped intensity support (Systems of Support to Improve Colorectal Cancer Screening and Follow-up Trial, SOS, R01CA121125, Green) doubled CRC screening uptake *(Annals of Internal Medicine 2013)*[2]. SOS showed a 30% net increase in fecal testing in year 1, and a 26% increase in adherence to screening over both years 1 and 2 (primary outcome, Table 1).

Adherent to CRC screening both years 1 and 2[a] (Primary Outcome)	Usual Care N=1166	Automated (Auto) N=1169	Auto+ Assisted N=1159	Auto + Assisted Navigated N=1170
All Participants	26%	51%*	58%*	65%*
Race/Ethnicity				
White (82%)	29%	51%*	58%*	67%*
Non-white race or Hispanic (18%)	17%	50%*	56%	56%
Never Screened Prior to Study				
No	19%	36%*	44%	54%
Yes	33%	64%*	70%	74%

[a] Adherence to CRC screening over 2 years is defined as completion of fecal tests in both years 1 and 2, flexible sigmoidoscopy or colonoscopy in year 1, or fecal occult blood test in year 1 and a flexible sigmoidoscopy or colonoscopy in year 2

*Comparisons to usual care and the less intensive arm(s) are significant at $P < 0.001$

The mailed fecal test program (Automated) was effective for all subgroups, defined by race/ethnicity, age, and education. However, among groups that received stepped-intensity interventions, increased-intensity interventions (phone assistance or this plus nurse navigation) had less or no impact on the subgroup that had never had a CRC test prior to study enrollment and in non-white racial or Hispanic ethnicity groups compared to whites. The stepped-intensity interventions that were not as effective in these subgroups were additional brief telephone assistance by a medical assistant (Assisted) for participants who did not complete screening after the mailed intervention alone, and nurse navigation (Navigated), which was ongoing telephone support until a fecal test or colonoscopy was completed. The proposed supplement will enhance the SOS study by providing evidence about whether adding financial incentives to the mailed intervention increases screening in harder-to-reach populations.

In year 6 of the SOS continuation (awarded in 2013), we interviewed participants who received at least three mailed fecal tests but did not complete screening[3].). The most common reason for not screening was related to not paying attention, either by not remembering, not making time for testing, or procrastinating ("out of sight, out of mind"). Other reasons were discomfort with handling stool, putting stool in the mail, and difficulty with dietary restrictions (e.g., in SOS year 3, KPWA* and SOS switched from a three-sample guaiac test to a single-sample FIT that requires no dietary changes). We also interviewed patients who recently completed their first fecal test, most often the FIT. New users noted: "it was easy to get it done." Based on our findings, in year 6 of SOS, we added back the nurse navigator (automated mailings + navigation) to address concerns and personalize screening. However, nurse navigation is expensive compared to automated mailings. If inattention is the primary reason for not testing or not trying the new test without dietary restrictions, financial incentives might help increase CRC screening in harder-to-reach populations. We propose testing this hypothesis.

We know of only one study that tested the comparative effectiveness of financial incentives for patients who complete fecal testing. Kullgren et al[4] conducted a study at a Veterans Administration (VA) clinic in Philadelphia, enrolling 1549 patients due for CRC screening in a two-part study. In part 1, patients were assigned to usual care or to receive $5, $10, or $20 for fecal occult blood test (FOBT) completion. In part 2 (based on the results of part 1), patients were assigned to usual care or to receive $5, $10, a 1 in 10 chance for $50, or entry into a $500 raffle for FOBT completion. Three sample guaiac fecal kits were handed out in clinic and included a brightly colored index card with the pre-assigned incentive for completion. No significant differences in FOBT completion were seen between the groups, except for the group assigned a 1 in 10 chance for $50. FOBT completion at 30 days was 49.3% versus usual care 29.7% ($P < 0.001$). Limitations included participation by men only (>99%), use of an older thee-sample test, and lack of comparisons by subgroups.

In this study, we will test behavioral economics principles that a probabilistic financial incentive (1 in 10 chance for $50) is still more effective than a guaranteed incentive of $10 alone. This comparison is important because a lottery program would be less expensive. Our study will determine if the lottery approach is generalizable to a non-VA male and female population, leading to an incremental increase in screening when added to a mailed fecal testing program (in contrast to the Kullgren study, which was clinic-based). It will also investigate differences by race/ethnicity and Medicaid coverage.

Information is limited on the effectiveness of financial incentives to increase uptake of other types of recommended cancer screening. The Community Preventive Services Task Force performed a systematic review of studies of client incentives to increase breast, cervical, and CRC screening uptake (search period 1996 to 2008) and found only one study.[1] Slater et al[5] randomized 51,226 women aged 50-64 living in low-income areas to either mailed, mailed plus incentive, or control groups. The mailing included a number to call to get a free mammogram and the incentive was $10 for completing the mammogram. Mammography uptake was very low among all three groups, but compared to the control group (1.7%), uptake was higher in the mailed group (2.0%), with mailed plus incentive statistically significantly higher (2.7%) than both control and mailed-only groups. In a more recent study, Merrick et al,[6] compared usual care to incentives of a $15 gift card, entry into a lottery for a $250 gift card, or choice of either the $15 card or the lottery; no differences in mammogram screening uptake were observed. Giles et al,[7] in a systematic review and meta-analysis, found 16 comparative effectiveness studies testing financial incentives for smoking cessation (10), vaccine uptake (4), physical activity (1), and breast cancer screening (1, Slater).[5] The analysis found that financial incentives were more effective than usual care or no intervention (relative risk 1.92, 95% confidence interval 1.46-2.53). We conclude that financial incentives appear to be effective for promoting health behaviors; however very little evidence is available on its effect on cancer screening and the Community Preventive Services Task Force recommends more research.[8]

Qualitative studies provide some information on the acceptability of financial incentives for encouraging healthy behaviors in general (e.g., tobacco cessation, weight loss). Giles et al[9] in a separate review of 81 papers and found that in general, financial incentives were considered acceptable, with five themes emerging: fair exchange (participation is voluntary); design (positive rewards, and not too large); effectiveness (evidence-based, with costs and resources required considered); recipients (populations targeted, certain incentives might be misused, e.g., to buy more cigarettes); and impact on society (people who need the incentive the least might be most likely to benefit, equality). In a recently published focus group study, some participants found financial incentives offensive, "rewarding people for the behaviors they should already have." However, ethicists and disparities researchers note that disadvantaged groups often need additional help to achieve health equity.[10]

Low utilization of screening leads to downstream disparities, delayed detection of CRC, diagnosis at more advanced stages, and higher CRC morbidity and mortality.[11]

4. Study Design

We propose to conduct a 3-arm randomized controlled trial to compare the effectiveness of an automated mailed fecal-testing program (**Arm 1 Mailed**) to two types of conditional monetary incentives provided upon completion of CRC testing: (1) a guaranteed financial incentive ($10) (**Arm 2** Mailed plus **Monetary**) or a probabilistic 1 in 10 chance of $50 (**Arm 3** Mailed plus **Lottery).** Our primary outcome will be completion of CRC testing at 6 months (defined as any type of recommended CRC test: FIT, colonoscopy, or flexible sigmoidoscopy). The primary analysis will test for difference in completion rates among randomization groups. Secondary analyses will explore differences (effect modification) in the effect of incentives on the primary outcome by race, ethnicity, and Medicaid subgroups.

Patients aged 50-75 as of January 1, 2017 and enrolled in 1 of 21 Western Washington KPWA Medical Centers will be eligible if they are not current for CRC screening, defined as no evidence in electronic health data (EHR and claims data) of fecal testing in the prior 12 months, no colonoscopy in the prior 9 years, and no flexible sigmoidoscopy in the prior 4 years. The clinics have more than 110,000 age-eligible patients and more than 34,000 are not current for CRC screening. Patients with a history of CRC or inflammatory bowel disease will be excluded. We will also exclude patients already participating in the ongoing SOS trial.

We will oversample from non-white and Hispanic groups and those with Medicaid coverage to ensure that at least two-thirds of the sample meets one or both of these criteria. KPWA has race and ethnicity data on 93% of patients in this age category, with almost 10,000 non-white or Hispanic patients who are overdue for CRC screening. At KPWA, almost 72% of white members aged 51-75 are current for CRC screening, while comparative rates among Asian, Black, and Native American members are 66-69%, with lower rates among Hispanic (64%) and Pacific Islander (61%) members, and patients with Medicaid insurance (55%).

We will send letters to 5000 potentially eligible patients describing the study with a brief survey to collect sociodemographic information and confirm eligibility. Questions will be from the main SOS study and are based on the Preventive Health model that predicted screening in our main SOS and other studies,[12, 13] asking about self-efficacy, pros and cons of fecal testing, social influence, and “defensive information processing” (e.g., “I like to ignore the fact that I could get cancer”), which specifically addresses avoidance and inattention to screening.[14] We will include a $2 bill with the survey (based on evidence that this increases participation rates)[15] to thank recipients for their time.

Based on our prior SOS ARRA Supplement (testing adherence to 3 types of fecal test kits),[16] we expect 29% to return the survey and 23% to be eligible and enrolled, with a target enrollment of 1150 participants (two-thirds non-white or Hispanic and one-third Medicaid [white or non-white]). We will use a computer-based randomized sequence generator to assign patients to: (1) Mailed; (2) Mailed plus $10; or (3) Mailed plus Lottery. Randomization will be stratified by history of prior CRC testing (yes/no based on electronic data), white vs. non-white race or Hispanic, or Medicaid status (yes/no) to ensure balance by these key characteristics. The randomization sequence will be concealed to everyone except the study programmer. Outcomes will be blinded until data collection is complete.

A second set of 5000 potentially eligible patients will receive mailings as described. Additionally, Kaiser Permanente Washington Health Research Institute’s (KPWHRI) Survey Research Program staff will conduct one reminder call to patients who do not return a survey after 14-21 days (approved by the KPWHRI Institutional Review Board on 6/12/2017). When respondents are available, the caller asks if they received the survey and if they have any questions. The patient may request another packet be mailed or refuse participation in the study at that time. The caller will ask for the survey to be returned within two weeks. When another person answers but the patient is not available, the caller leaves a message that they are calling from KPWHRI about a study and requests the best time of day to call again. If the caller receives an answering machine, a voicemail is left with a phone number to request a new questionnaire.

5. Study Population

a. Proposed Number of Subjects

	Invited (contacted)	Enrolled
Kaiser Permanente patients	10,000	1150
Other sites	0	0
Total	10,000	1150

b. Inclusion and Exclusion Criteria

<u>Inclusion Criteria</u>

KPWA patients aged 50–75 as of January 1, 2017, continuous enrollment of at least one year, and receiving care in one of the 21 KPWA Medical Centers.

<u>Exclusion Criteria</u>

Individuals will be excluded if they have:

- Completed FIT in the prior 12 months (automated EHR data) or colonoscopy in the prior 9 years (automated EHR or claims data, and self-report per survey).
- A history of CRC (automated data or survey).
- A history of total colectomy, inflammatory bowel disease, end stage renal failure, end stage liver disease, dementia, hospice, nursing home residence based (automated data).
- A family history of parent, brother, sister, or child (blood relative) diagnosed with colorectal cancer at age 60 or younger (self-report survey).
- Participated in the SOS main trial.

Data collected for recruitment purposes will be kept on both participants and non-responders to determine the representativeness of study participation. Data include age, insurance type, race/ethnicity, sex, and census block data. No data will be kept on individuals refusing participation (calling the research number to opt out).

c. Vulnerable Populations

None

d. Setting

Participants will be recruited by mail only.

e. Recruitment Methods

The study programmer will identify eligible subjects using KPWA virtual warehouse data.
The KPWHRI Survey Research Program will mail an invitation letter, information sheet and survey to potentially eligible patients. The letter will include a $2 incentive. Participants will be recruited after they return their survey and the study team confirms their eligibility.

In the second wave of recruitment, patients who do not return a survey will be given a reminder call 14-21 days after being sent a survey.

f. Consent Process

Participants will receive an information sheet written in plain language explaining the study in detail along with the invitation letter and survey. Participants who agree to participate will complete and return the baseline survey.

g. Non-English-Speaking Participants

Since we are targeting patients who are non-white or Hispanic or have Medicaid insurance, non-English speakers may obtain assistance in filling out and returning the survey. We will not exclude these patients

because we will not know they do not speak English. Participants will receive wordless instructions for the FIT test,[17] which may help individuals with low literacy or who are non-English speakers complete fecal testing.

h. HIPAA Privacy Rule Authorization – if study will use or disclose Protected Health Information

Identifying participants is not practical without a waiver of consent because this is the only way to identify participants to include them. Identify participants and proactively inviting them to participate in the study would be impossible without accessing individual-level contact information and data on their colon health.

The waivers of consent will not adversely affect the rights and welfare of potential participants. They will not be denied access to any benefit or service as a consequence of participation or non-participation. Furthermore, safeguards are in place to protect the confidentiality of all potential research participants.

We believe the minimal risk to privacy posed by our methods is outweighed by the benefits of this research to the scientific field and to individual participants' and populations' health.

6. Study Procedures

7. The study programmer will identify eligible using KPWA virtual warehouse data.
   - The study programmer will identify eligible subjects using the KPWA virtual warehouse data
   - The KPWHRI Survey Research Program will mail an invitation letter, information sheet describing the study (Page 11), survey (Page 14), and a $2 incentive to potential participants.
   - If interested, potential participants return the survey in a-postage paid envelope to the Survey Research Program.
   - In the second wave of recruitment, patients who do not return a survey will be called by Survey staff 14-21 days after receiving the survey to remind them to return it.
   - The Survey staff confirm eligibility of individuals who return their survey. We will not follow up with people who return a survey and are found to be ineligible.
   - The study programmer will perform randomization using a concealed automated computer-generated program with participants assigned to one of three intervention groups.
   - Intervention groups

     Group 1: Survey staff will mail participants a pamphlet on CRC testing choices and a letter stating that they will soon receive a FIT kit in the mail, and a number to call if they prefer another option. One week later, Survey staff will mail a second letter, FIT kit, pictograph, wordless instructions, and a postage-paid return envelope. A reminder letter will be sent to participants not completing the FIT kit after 3 weeks.

     Group 2: These participants receive the same intervention as group 1 with the following modifications: Mailing 1, the letter will include <u>notification that they will receive $10 (cash)</u> as a thank you for completing testing (FIT, flexible sigmoidoscopy, or colonoscopy) within 6 months. Mailing 2 (FIT kit with letter and wordless pictograph instructions) and mailing 3 (reminder letter for those still not returning kits) will include the same information about the $10 thank you for CRC screening completion. Mailing 4 will include a thank you and $10 for those who complete screening.

     Group 3: These participants receive the same intervention as group 1 with the following modifications: Mailing 1, the letter will include <u>notification that they will have a 1 in 10 chance of winning $50</u> for completing testing (FIT, flexible sigmoidoscopy or colonoscopy within 6 months). Mailing 2 (FIT kit with letter and wordless pictograph instructions) and mailing 3 (reminder letter for those still not returning kits) will include the same information about the 1 in 10 chance of winning $50 for CRC screening completion. Mailing 4 will include a thank-you letter to all and $50 to the

lottery winners. At the end of 6 months, we will collect the following participant data from electronic records: CRC screening status, preventive visits (Pap smear last 3 years and mammogram last 2 years for women), BMI, Charlson co-morbidity score, utilization, primary care visits, and preventive visits related to colon health.

Data Collection: We will collect the following information from electronic health records and participants.

EHR data and use	Sample	Sample Socio-demographic*	Stratify Variable	Participants	Date Range
Age (50 and <75 as of 1/1/17)	X	X	X	X	As of 1/1/17
Birthdate	X				As of 1/1/17
CRC diagnosis	X				Ever
Inflammatory Bowel Disease	X				Ever
Dementia	X				2016
End-stage liver failure	X				2016
End-stage renal disease, Stage 4 or Dialysis	X				2016
Hospice	X				2016
Clinic	X		X	X	As of 1/1/17
Insurance type	X	X	X	X	Last recorded
Race/ethnicity	X	X	X	X	Last recorded
CRC screening status ever			X	X	All years enrolled
Fecal test status	X		X	X	Until end of 2018“
Flex sig status			X	X	Until end of 2018
Colonoscopy status	X		X	X	Until end of 2018
Sex	X	X		X	As of 1/1/17
Census block data	x	x		X	As of 1/1/17
Pap smear last 3 yrs., women				X	2014-2016
Mammogram last 2 yrs. women				X	2015-2016
BMI				X	Last recorded
Charlson Co-morbidity	X			X	2016
Utilization, primary care visits, preventive visits				X	2017
* Data will be kept on participants and non-responders to determine the representativeness of study participation. No data will be kept on individuals refusing participation (calling the research number to opt out).<br>CRC = colorectal cancer; flex sig = flexible sigmoidoscopy; BMI = body mass index					

We will also collect basic demographic data on non-responders (Sample sociodemographic column above). We will not keep identifiers except for census block data. We will destroy all other identifiers as soon as enrollment is completed.

A study aim is to determine if financial incentives decrease screening disparities. We need to know the representativeness of participants to evaluate this. We will compare the sociodemographic characteristics of participants to non-participants to determine the generalizability of our results, particularly for characteristics that pertain to groups with known screening disparities (non-white race, Hispanic ethnicity, Medicaid insurance, and younger age, i.e., aged 50-64)

Weekly, the SOS Disparities programmer will look for enrolled participants who have completed a FIT, flexible sigmoidoscopy or colonoscopy in the previous week. The study programmer will notify Survey Research Program

staff of results. Survey staff will then mail the participant an incentive based on their group assignment. A participant will have up to 6 months to complete a CRC screening test in order to receive an incentive.

We will not recontact people who return the survey and are ineligible and not collect any additional data from KPWA records beyond what we collect to identify the sample.

After all patients are randomized and have completed 6 months of follow-up, we will collect the following participant data from electronic records: CRC screening status, preventive visits (Pap smear last 3 years and mammogram last 2 years for women), body mass index, and diagnoses for calculating Charlson Co-morbidity scores. We will collect and keep data as described in the protocol on all enrolled participants, even if the participants choose not to return their FIT kit.

8. Data Analysis

Analysis Plan

Primary Outcome: The primary outcome is CRC screening within the 6 months following randomization (yes/no). We will use electronic data to measure outcomes, which we previously demonstrated as highly valid for accurately capturing CRC tests.[18] We will report the number and percent screened by randomization group. Generalized linear models with logit link and robust variance estimator will estimate the probability of screening. We will use predictive margins based on these logistic models to estimate differences in screening rates between randomization groups. Models will adjust for age, sex, race/ethnicity, education, and screening history prior to randomization. With a targeted enrollment of 1150 participants, we will have 80% power to detect a 10% net difference in CRC screening uptake among randomization groups.

Secondary Analyses will explore whether the effect of incentives on screening uptake differs by race/ethnicity, Medicaid coverage, income, education, sex, and history of prior CRC testing. Interaction terms between these variables and the intervention group will be included in multivariable models, and Wald tests will be used to assess the overall significance of effect modification.

Limitations and Strengths:

- Although we have sufficient power to test our primary outcome, overall differences in CRC screening uptake at 6 months for either Monetary or Lottery incentives compared to Mailed only, we will not have sufficient power to test differences by subgroup (race, ethnicity, low income). We will be able to compare the magnitude of effects by subgroups and generate hypotheses for further study.
- All patients have health insurance and, similar to the Northwest, the overall population is primarily white. However, we will assure adequate representation of disparity populations (at least two-thirds) by oversampling from non-white and Medicaid patient populations.
- Intervention materials will be designed for low levels of literacy (including the use of wordless FIT test instructions developed for the STOP CRC study ([UH3CA188640, Coronado, Green]),[17] but not culturally tailored to specific race, ethnicity, and language groups. Subsequent studies could explore this further.
- Offering financial incentives sometimes raises ethical concerns, especially when specifically offered to disadvantaged populations. Yet we argue that we are studying a health equity issue and some groups need additional support to achieve the same health outcomes of more advantaged groups. We work with several Medicaid insurance providers (not within KPWA) who have been providing monetary incentives to patients for completion of fecal testing, but who do not know if this strategy is effective. We have consulted our Institutional Review Board. They reviewed the study design and had no objections.
- Our study is ambitious given the limited funding provided by the supplement. Because of the parent grant we will be able to efficiently recruit, implement, and evaluate the proposed study. We received an ARRA administrative supplement (CA121125-A2, Green) with similarly ambitious goals, and achieved our targeted enrollment and completed all study aims with results published promptly at the end of the funding period. [16, 19, 20]

9. Privacy, Confidentiality, and Data Security

Data will be collected in KPWA computerized data systems in accordance with laws governing confidentiality of medical records and HIPAA requirements. All employees at KPWA sign agreements to maintain confidentiality of data and research information. All computers in KPWHRI require passwords to access the network and the electronic mail system.

Automated data and identifying information volunteered by participants (name, telephone number, medical record number) will be collected and stored on a secure server at KPWHRI. Only study investigators and study staff will have access to these data. Patients will be assigned a unique, study-specific identification number, and all datasets that will be used for analyses will be stripped of any identifying data, excluding dates, and thus be a limited dataset. Thus, the final analytic database will include unique participant codes and dates but no other identifying data.

Identifiers will be destroyed no later than 5 years after the completion of the study, or by June 2023.

10. Provisions to Monitor the Data to Ensure the Safety of Participants

Participants may change their mind at any time about letting us use their information for this study. If they change their mind, they may take back their consent by writing to the principal investigator. Taking back their consent will not affect their health care or benefits at KPWA. We may still use the study information we collected before we received the letter taking back their consent.

There are no anticipated circumstances under which participants will be withdrawn from the research without their consent.

The study FIT tests are ordered by Dr. Green but results are sent to the patient's provider. KPWA uses an algorithm for assigning patients to a provider at enrollment, so all patients have a physician or a midlevel provider at their assigned clinic. Providers and their teams will review FIT tests per usual workflow (negative tests are reviewed by the team and positives by the patient's provider).

Follow-up of abnormal stool tests will use the same procedures as the original SOS study. SOS study positive FIT results are managed the same way as KPWA positive FIT tests:

1. The positive study FIT automatically is listed in the patient's provider laboratory inbox.
2. The patient automatically gets a notification of the positive FIT test (letter or their MyChart inbox or both) and a letter notifying them to call their provider's team about the test result if they have not already been contacted.
3. The positive study FIT is automatically tracked by a KPWA positive FIT test registry, which reminds providers if a colonoscopy is not completed, or has the provider fill out a form why a colonoscopy was not done. Providers are required to comply with this process or their medical center chief is notified.

11. Risks and Benefits

a. Risks to Participants

The potential risk to participants is breach of confidentiality. Participants with a positive FIT may experience worry related to the test results.

b. Potential Benefits to Participants

The direct benefit to participants is potential improvement in physical health due to completing CRC screening. The indirect benefit is an increase in scientific knowledge for society.

12. Costs to Participants

There is no cost to the participant for completing the FIT kit.

13. Compensation to Participants

Participants will receive an incentive of $2 in their invitation letter. Participants in Group 2 who complete a CRC screening test will receive $10. Participants in Group 3 will have a 1 in 10 chance of receiving $50 for completing a CRC screening test.

14. Flow Diagram

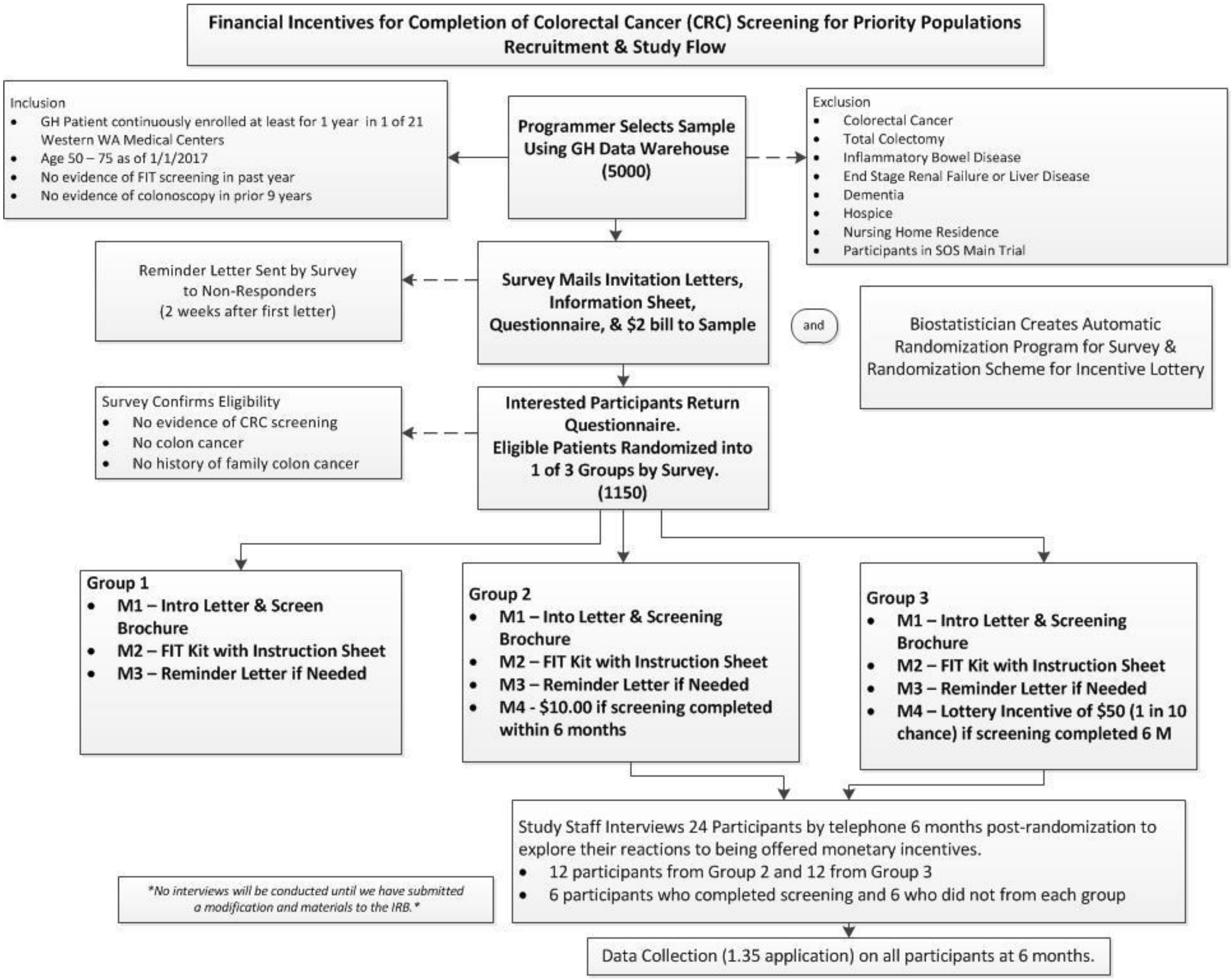

14. Consent information

Kaiser Permanente Washington Health Research Institute (KPWHRI)

Smart Options for Screening Study **Information Sheet**

Researchers

KPWHRI	Consultants
Beverly Green, MD, MPH<br>Physician & Lead Researcher<br>(287-2997)	Jeffrey T. Kullgren, MD, MS, PMH<br>University of Michigan Medical School
Jessica Chubak, PhD<br>Co-Lead Researcher (287-2556)	Laura Aubree Shay, PhD, MSSW, LCSW,<br>University of Texas School of Public Health

We are asking you to be in a research study. This form explains the details of the study.

Please take as much time as you need to read through this information and make your choice. Call our **toll-free study line at 1-800-206-0445**, if anything isn't clear or if you would like more information.

You do not have to be in this study. If you say yes, you can quit the study at any time.

What is this study about?

Screening is a way to test for cancer before symptoms show up. It can save lives by finding cancer before it spreads. We are trying to learn the best ways to support people in getting screened for colon cancer. The purpose of the study is to learn if financial incentives change screening rates and if there are differences by ethnicity/race, Medicaid coverage, income, education, sex, or history of prior screening.

What are you asking me to do?

We are asking you to fill out and return the enclosed survey to us in the postage paid envelope. The survey asks you questions about your colon health, screening history and knowledge about colon cancer. It will take about fifteen minutes to fill out. You can skip any questions you do not want to answer.

In order to participate in the study, the survey needs to be completed and returned to us within sixty days. Study staff may call you within a few weeks to make sure you received the survey. Please try to fill out the survey as soon as possible. Once we receive your survey, we will review it to make sure you are still eligible to be in the study.

Even if you are not eligible to be in the study, please return your survey to us in the postage paid envelope so we have a count of the number of people who are ineligible.

What will happen if I take part in this study?

If you are eligible, we will by chance, like a roll of the dice, assign you to one of three groups.

No matter what group you are in, we will send you the following materials:

- A letter and brochure about colorectal screening options.
- A FIT screening kit along with instructions on how to use it.

We will ask you to complete your FIT or other colon cancer screening test (colonoscopy or flexible sigmoidoscopy) as soon as you can. If you complete your screening test within six months, you may receive money depending on which group you are in.

We will use a computer to collect information about your use of health care services related to colon health, including:

- Lab test results and procedures related to colon health
- Number of chronic conditions
- Other screening tests (mammography, pap smears)
- Use of health care services (primary care and preventive health visits).

We will also collect information on clinic, age, sex, race, insurance type, height, weight, and census information, like education and income level, about the neighborhood you live in. We will collect this information from the time you enter the study through the end of 2023 on all enrolled participants even if you choose not to complete any colorectal cancer screening. Once you return the enclosed survey to us, if you are still eligible, you will be enrolled in the study.

Will there be any costs to me?
There will not be any cost to you for completing your FIT screening test.

Will being in this study help me?

We hope the results of this study will improve screening for colon cancer at Kaiser Permanente and in other health systems around the country.

Can anything bad happen to me from being in this study?

You might feel uncomfortable answering some study questions. A positive FIT test may create stress for some people. You may skip any questions you don't want to answer.

It's possible that someone other than the researchers could find out you were in the study or see your private study information. The steps we take to keep this from happening are described below.

How will you protect my confidentiality?

All Kaiser Permanente researchers sign a confidentiality pledge that requires them to keep your information private.

The researchers listed on the first page and their staff will use your study information for research only. We won't use your name in study reports or write it on your study information. Instead, we will label everything with a code number only. We won't tell your doctor whether or not you join this study.

We plan to keep your health information as described in this form until 2023. At that time, we will destroy any study records that include your name or other information that points to you. Any study test results in your personal medical record will not be deleted.

How does HIPAA apply to this study?

Your health information is protected by a federal privacy law called HIPAA. Kaiser Permanente must follow this privacy law. According to HIPAA, the information collected by the researchers for this study is part of that protected health information. HIPAA requires that the researchers tell you the following:

By being in this study, you are giving Kaiser Permanente permission to allow the researchers to collect, use, and share the following information about you for this study:

1. Your survey answers.
2. Your medical record information as described above.

It is possible that staff from Kaiser Permanente and the funding agency may look at our study records for oversight. We will not share the information we collect for this study with anyone else except as allowed by law.

The HIPAA privacy law does not always apply to those who are given protected health information. Once Kaiser Permanente has given out health information, the person who receives it may re-disclose it. Privacy laws may no longer protect the information.

In order to be in the study, you must agree to this use of your health information.
The permission for the researchers to obtain your health information for this study ends in 2023.

Do I have to be in this study?

No, being in this study is up to you. You are free to say no now or to leave the study at any time later. Either way, there will be no penalty. Your decision won't affect the health care you receive or benefits that you are entitled to.

What happens if I say yes, but change my mind later?

You may change your mind any time about letting us use your information for this study. If you change your mind, you may take back your consent by writing to:

> Bev Green, MD, MPH
> Kaiser Permanente Washington Health Research Institute
> 1730 Minor Ave, Suite 1600
> Seattle, WA 98101

If you take back your consent, it will not affect your health care or benefits at Kaiser Permanente. We may still use the study information we collected before we received the letter taking back your consent. But we will destroy any record of your name or other information that could identify you.

Who do I call if I have questions?

If you have questions or concerns about the study, please call us **toll free at 1-800-206-0445**

If you have questions about your rights as a research participant, please call the Kaiser Permanente Washington Human Subjects Review Office at 206-287-2919.

15. Smart Options for Screening Survey

Survey # (preassigned) **Date Completed: _______**

These questions are about your history of colorectal screening. You can skip any question you do not want to answer.

By returning this survey, I agree to participate in the Smart Options for Screening (SOS) program as described in the Information Sheet.

Please read each question carefully and put an x in the box that best describes your answer.

<u>The stool blood test</u>, also known as a fecal occult blood test, is done at home to determine whether the stool contains blood. A sample of your fecal matter or stool is collected from 1 bowel movement and then the sample is returned in a small tube to a lab to be tested.

1. Have you ever done a stool blood test at home?

□ Yes
□ No

The next 2 questions ask about two other screening tests called a <u>sigmoidoscopy and colonoscopy</u>. These are tests that check for colon cancer. Both tests examine the colon using a narrow, lighted tube that is inserted in the rectum. Sigmoidoscopy only examines the lower part of the colon, while colonoscopy examines the entire colon.

With the sigmoidoscopy:

- Only the lower part of the colon is examined.
- You are awake.
- You are able to drive yourself home.
- You are able to resume normal activities after the test.

With the colonoscopy:

- The entire colon is examined.
- You are given medicine through a needle in your arm to make you sleepy.
- You need someone to drive you home.
- You need to take the rest of the day off from normal activities.

The following question is about <u>colonoscopy</u>. A colonoscopy examines the entire colon using a narrow, lighted tube that is inserted in the rectum. With a colonoscopy, you are given medicine through a needle in your arm to make you sleepy, you need someone to drive you home, and you may need to take the rest of the day off from your usual activities.

2. Have you ever had a -<u>*COLONOSCOPY*</u>?

□ Yes
□ No

3. When was your MOST RECENT COLONOSCOPY?

□ 9 years ago, or less → **Go to instruction box on next page**
□ 10 years ago or more Continue

The following question is about the <u>sigmoidoscopy</u>, also referred to as flexible sigmoidoscopy or "flex sig". Sigmoidoscopy examines only the lower part of the colon. You are awake during the test, can drive yourself home, and can resume normal activities after the test.]

4. Have you ever had a *<u>SIGMOIDOSCOPY</u>*?
□ Yes
□ No

5. Have you ever been diagnosed with colorectal (colon) cancer?
□ Yes → **Go to instruction box below**
□ No Go to Question 6

6. Do you have a parent, brother, sister, or child (blood relative) who was diagnosed with colorectal cancer <u>at age 60 or younger?</u>
□ Yes → **Go to instruction box on (page x/below)**
□ No Go to the top of Page 3
(this will be boxed)

<u>If you answered</u>

- *<u>That you had a colonoscopy 9 years ago or less,</u>*
- *<u>That you have ever been diagnosed with colorectal (colon) cancer, or</u>*
- *<u>That you have a blood relative who was diagnosed with colorectal cancer at age 60 or younger,</u>*

You are not eligible to be part of the study. We appreciate your taking time to answer these few questions. ***Please place the survey in the postage paid envelope and mail it back to us,*** *it's important for us to know why people are not eligible to complete the full survey Thank you for your time and interest.*

The next few questions ask what **you think** your chances are of getting colon cancer. There is no right or wrong answer.

7. Compared with other people my age, I think MY chance of developing colon cancer in the next 10 years is...
□ Much lower than average
□ A little lower than average
□ About average (50/50)
□ A little higher than average
□ Much higher than average

8. Considering your life right now, how important is it for you to get screened for colon cancer in the next 6 months?
□ Very important
□ Somewhat important
□ Not very important
□ Not at all important

People can have many different reasons why they do not want to get screened for colon cancer. On a scale from 1 to 5, please tell us how much you agree or disagree with the following reasons for not getting screened for colon cancer.

It would be difficult for me to be screened for colon cancer because…

Strongly Disagree → Strongly Agree

9. The test might find something wrong.	1	2	3	4	5
10. It is too embarrassing.	1	2	3	4	5
11. The stool blood test might be disgusting.	1	2	3	4	5
12. There might be unexpected costs or bills to pay.	1	2	3	4	5
13. I have other priorities right now, but I will eventually get colon cancer screening.	1	2	3	4	5
14. I don't think I need it because I don't think I will get colon cancer	1	2	3	4	5

Now we would like to know what would make you **want** to get screened for colon cancer. On a scale from 1 to 5, please tell us how much you agree or disagree with the following reasons **to get screened** for colon cancer.

I want to get screened for colon cancer because …

Strongly Disagree → Strongly Agree

15. Finding cancer early gives me a better chance at a cure.	1	2	3	4	5
16. Receiving clear findings would give me peace of mind.	1	2	3	4	5
17. My family would be happy if I got screened.	1	2	3	4	5
18. Getting screened is part of taking care of myself.	1	2	3	4	5
-19. The benefits of getting screened outweigh the difficulties.	1	2	3	4	5
20. If I get screened, I will be less likely to get colon cancer	1	2	3	4	5

These next questions ask how confident you are about different aspects of colon cancer screening. Please use a scale from 1 to 5, with 1 being "Not at all confident" and 5 being "Very confident" to answer these questions.

How confident are you that you can…

	**Not at all confident**				**Very confident**
21. Find time to complete colon cancer screening?	1	2	3	4	5
22. Talk to your doctor about colon cancer screening?	1	2	3	4	5
23. Carry out any necessary steps to complete colon cancer screening?	1	2	3	4	5
24. Get support from family and friends to help you complete colon cancer screening?	1	2	3	4	5

For the next set of statement, please indicate how much you agree or disagree. There are no right or wrong answers. Try not to let your answer to one statement influence your answers to other statements.

	**Strongly Disagree**						**Strongly Agree**
25.. I don't see the point of going to a doctor unless I'm really sick.	1	2	3	4	5	6	7
26.. I don't go to the doctor unless it's really serious.	1	2	3	4	5	6	7
27. If I feel healthy I do not go to the doctor for a routine check-up.	1	2	3	4	5	6	7
28. I can't do everything I am supposed to do for my health it'd be a -time job.	1	2	3	4	5	6	7
29. In uncertain times, I usually expect the best.	1	2	3	4	5	6	7
30. If something can go wrong for me, it will.	1	2	3	4	5	6	7
31. I'm always optimistic about the future.	1	2	3	4	5	6	7
32. I hardly ever expect things to go my way.	1	2	3	4	5	6	7
33. I rarely count on good things happening to me.	1	2	3	4	5	6	7
34. Overall, I expect more good things to happen to me than bad.	1	2	3	4	5	6	7

For each of the statements below, please indicate whether the statement is like you. Please mark "1" for "Not at all like me" and "5" for "Very much like me". If you fall somewhere in between, please mark the number that is truest for you.

	**Not at all like me (1)**	**Somewhat not like me (2)**	**Uncertain (3)**	**Somewhat like me (4)**	**Very much like me (5)**
35. I only act to take care of immediate concerns, figuring the future will take care of itself.	1	2	3	4	5
36. I make decisions or take actions based on how easy there are to do.	1	2	3	4	5

38. Overall, thinking of your assets, debt and savings, how satisfied are you with your current financial condition? Please use a 1 to 10 scale, where 1 means "Not at all satisfied" and 10 means "Extremely Satisfied".

Not at all Satisfied									Extremely Satisfied
1	2	3	4	5	6	7	8	9	10

39. How much do you worry about being able to pay your medical bills?

☐ A lot
☐ A little
☐ Not at all

The next question is about your general health.

40. In general, would you say your health is:

☐ Excellent
☐ Very good
☐ Good
☐ Fair
☐ Poor

41.Do you smoke tobacco every day (including cigarettes, e-cigarettes, cigars, pipes)?

☐1 Yes ☐2 No ☐3 Don't know

These last questions are about you. Your answers will be combined with all answers.

42. Do you consider yourself to be Hispanic or Latino?

☐ Yes
☐ No

43. What race do you consider yourself to be? (Record all that apply)

- ☐ American Indian /Alaska Native
- ☐ Asian
- ☐ Native Hawaiian or Pacific Islander
- ☐ Black or African American
- ☐ White
- ☐ More than One Race

44. How often do you need to have someone help when you read instructions, pamphlets, or other written materials from your doctor or pharmacy?

- ☐ Never
- ☐ Rarely
- ☐ Sometimes
- ☐ Often
- ☐ Always

45. What is your current marital status?

- ◻ Married
- ◻ Living with partner
- ◻ Single/never married
- ◻ Widowed
- ◻ Divorced
- ◻ Separated

46. What is the **highest** level of school that you have completed or the **highest** degree you have received?

- ◻ Less than high school
- ◻ High school degree or GED
- ◻ Some college, no degree
- ◻ Bachelor's degree
- ◻ Master's/Professional/Doctoral degree

47. What best describes your current paid work status? Please select one

- ☐ Working full-time
- ☐ Working part-time
- ☐ Retired
- ☐ Not working

48. During the past year, about how much money do you estimate members of your immediate household received from all sources such as work, Social Security, interest, investments, etc.? *Your best guess is fine!*

- ☐ Less than $10,000
- ☐ Between $10,000 and $20,000
- ☐ Between $20,000 and $50,000
- ☐ Between $50,000 and $100,000
- ☐ More than $100,000

REMINDER CALL
CONTACT SCRIPT (abridged)

Contact
Hello. This is [INTNAME] calling from Kaiser Permanente Washington Health Research Institute.
May I please speak with (RESPONDENT)?

RESPONDENT NOT AVAILABLE

Hello, this is [INTNAME] and I'm calling from Kaiser Permanente Research Institute about a study.
Generally, are mornings, afternoons or evenings the best time to reach [RESPONDENT]?

RESPONDENT AVAILABLE

Hello, this is [INTNAME] and I'm calling from Kaiser Permanente Research Institute.
Recently we sent you a survey in the mail asking questions about colon cancer screening
We haven't received your survey, so we are calling to confirm that you got it and to see if you have any questions.
Did you receive the **survey**?
IF NEEDED: DESCRIPTION OF PACKET/ENVELOPE
Do you have any questions about the **survey** or the study?

We sent it in a large envelope with the Kaiser Permanente Washington logo on the upper left corner. The envelope contained a letter, information sheet about the study, the survey, and a large postage-paid return envelope.

1	YES/HAVEN'T RETURNED	PLS SEND
2	YES, ALREADY RETURNED	TKSEXIT
4	NOT SURE; REQUESTS ANOTHER	SKP SECLTR2
5	NO	SKP SECLTR2
8	R REFUSES	SKP THANKREF
9	SOME OTHER PROBLEM	EXIT

We will send you another **survey** within the week.

1	YES, SEND 2ND SURVEY	SKP UPDADDR
8	R REFUSES	SKP THANKREF
9	SOME OTHER PROBLEM	EXIT

VOICE MAIL SCRIPTS:

This is (INT NAME) calling from Kaiser Permanente Washington Health Research Institute.
I am calling for ___________
Today is ______________

Recently, we mailed you a survey and haven't received it yet.

If you have already returned **the questionnaire,** thank you.

If you have any questions about **the study or survey**, or, have not received the materials, please call us at 1-877-246-0559. Leave your name and phone number and someone will **call you back/send another survey**

1. Sabatino SA, Lawrence B, Elder R, Mercer SL, Wilson KM, DeVinney B, Melillo S, Carvalho M, Taplin S, Bastani R, Rimer BK, Vernon SW, Melvin CL, Taylor V, Fernandez M, Glanz K, Community Preventive Services Task F. Effectiveness of interventions to increase screening for breast, cervical, and colorectal cancers: nine updated systematic reviews for the guide to community preventive services. Am J Prev Med. 2012;43:97-118.
2. Green BB, Wang CY, Anderson ML, Chubak J, Meenan RT, Vernon SW, Fuller S. An automated intervention with stepped increases in support to increase uptake of colorectal cancer screening: a randomized trial. Ann Intern Med. 2013;158:301-11. PMCID: 3953144.
3. Green BB, BlueSpruce J, Tuzzio L, Vernon SW, Aubree Shay L, Catz SL. Reasons for never and intermittent completion of colorectal cancer screening after receiving multiple rounds of mailed fecal tests. BMC Public Health. 2017;17:531. PMCID: 5450259.
4. Kullgren JT, Dicks TN, Fu X, Richardson D, Tzanis GL, Tobi M, Marcus SC. Financial incentives for completion of fecal occult blood tests among veterans: a 2-stage, pragmatic, cluster, randomized, controlled trial. Ann Intern Med. 2014;161:S35-43.
5. Slater JS, Henly GA, Ha CN, Malone ME, Nyman JA, Diaz S, McGovern PG. Effect of direct mail as a population-based strategy to increase mammography use among low-income underinsured women ages 40 to 64 years. Cancer Epidemiol Biomarkers Prev. 2005;14:2346-52.
6. Merrick EL, Hodgkin D, Horgan CM, Lorenz LS, Panas L, Ritter GA, Kasuba P, Poskanzer D, Nefussy RA. Testing novel patient financial incentives to increase breast cancer screening. Am J Manag Care. 2015;21:771-9.
7. Giles EL, Robalino S, McColl E, Sniehotta FF, Adams J. The effectiveness of financial incentives for health behaviour change: systematic review and meta-analysis. PLoS One. 2014;9:e90347. PMCID: PMC3949711.
8. The Guide to Community Preventive Services. Client-Oriented Interventions to Increase Breast, Cervical, and Colorectal Cancer Screening. [cited April 26, 2016]. Available from.
9. Giles EL, Robalino S, Sniehotta FF, Adams J, McColl E. Acceptability of financial incentives for encouraging uptake of healthy behaviours: A critical review using systematic methods. Prev Med. 2015;73:145-58.
10. Chin MH. Creating the Business Case for Achieving Health Equity. J Gen Intern Med. 2016.
11. Siegel RR, Miller KD, Jemal A. Cancer statistics, 2016. CA Cancer J Clin. 2016;66:7-30.
12. Murphy CC, Verson SW, Haddock NM, Anderson ML, Chubak J, Green BB. Longitudinal predictors of colorectal cancer screening among participants in a randomized controlled trial. Prev Med. 2014;66:123-30. PMCID: 4209306.
13. Gordon NP, Green BB. Factors associated with use and non-use of the Fecal Immunochemical Test (FIT) kit for Colorectal Cancer Screening in Response to a 2012 outreach screening program: a survey study. BMC Public Health. 2015;15:546. PMCID: 4462185.
14. McQueen A, Vernon SW, Swank PR. Construct definition and scale development for defensive information processing: an application to colorectal cancer screening. Health Psychol. 2013;32:190-202.
15. Doody MM, Sigurdson AS, Kampa D, Chimes K, Alexander BH, Ron E, Tarone RE, Linet MS. Randomized trial of financial incentives and delivery methods for improving response to a mailed questionnaire. Am J Epidemiol. 2003;157:643-51.
16. Chubak J, Bogart A, Fuller S, Laing SS, Green BB. Uptake and positive predictive value of fecal occult blood tests: A randomized controlled trial. Prev Med. 2013;57:671-8. PMCID: PMC3856243.
17. Coronado G, Sanchez J, Devoe J, Green B. Advantages of wordless instructions on how to complete a fecal immunochemical test: Lessons from patient advisory council members of a federally qualified health center. Journal of Cancer Education. 2014;29:86-90. PMCID: 3946071.

18. Green BB, Wang CY, Horner K, Catz SL, Meenan RT, Vernon SW, Carrell D, Chubak J, Ko C, Laing S, Bogart A. Systems of support to increase colorectal cancer screening and follow-up rates (SOS): design, challenges, and baseline characteristics of trial participants. Contemp Clin Trials. 2010;31:589-603. PMCID: 2956868.
19. Laing SS, Bogart A, Chubak J, Fuller S, Green BB. Psychological distress after a positive fecal occult blood test result among members of an integrated healthcare delivery system. Cancer Epidemiol Biomarkers Prev. 2014;23:154-9. PMCID: 3947142.
20. Wang CY, de Dieu Tapsobsa J, Anderson ML, Vernon SW, Chubak J, Fuller S, Green BB. Time to screening in the systems of support to increase colorectal cancer screening trial. Cancer Epidemiol Biomarkers Prev. 2014;23:1683-8. PMCID: 4119537.
