# Supplementary material for: Financial Incentives to Increase Colorectal Cancer Screening Uptake and Decrease Disparities: A Randomized Clinical Trial
Source: JAMA Netw Open. Author manuscript; Available in PMC 2019 Oct 12. (PMC6789432; doi:10.1001/jamanetworkopen.2019.6570)
Supplement: Supplement 2 — eTable 1. Proportions Tested for Colorectal Cancer Within 6 Months From Sensitivity Analysis Excluding Participants Who Tested After Questionnaires Were Mailed but Before Randomization and Receiving Information About Incentives eTable 2. Subgroup Analyses of Intervention Effects on FIT Completion by Sociodemographic Characteristics With Separate Estimates of the Effects of Mail and Monetary and Mail and Lottery Interventions eTable 3. Subgroup Analyses of Intervention Effects on FIT Completion by Psychosocial Measures Self-Reported at Baseline With Separate Estimates of the Effects of Mail and Monetary and Mail and Lottery Interventions [file NIHMS1047899-supplement-Supplement_2.pdf]

Supplementary Online Content

Green BB, Anderson ML, Cook AJ, et al. Financial incentives to increase colorectal cancer screening uptake and decrease disparities: a randomized clinical trial. *JAMA Netw Open*. 2010;2(7):e196570. doi:10.1001/jamanetworkopen.2019.6570

eTable 1. Proportions Tested for Colorectal Cancer Within 6 Months From Sensitivity Analysis Excluding Participants Who Tested After Questionnaires Were Mailed but Before Randomization and Receiving Information About Incentives

eTable 2. Subgroup Analyses of Intervention Effects on FIT Completion by Sociodemographic Characteristics With Separate Estimates of the Effects of Mail and Monetary and Mail and Lottery Interventions

eTable 3. Subgroup Analyses of Intervention Effects on FIT Completion by Psychosocial Measures Self-Reported at Baseline With Separate Estimates of the Effects of Mail and Monetary and Mail and Lottery Interventions

This supplementary material has been provided by the authors to give readers additional information about their work.

© 2019 Green BB et al. *JAMA Network Open.*

eTable 1. Proportions Tested for Colorectal Cancer Within 6 Months From Sensitivity Analysis Excluding Participants Who Tested After Questionnaires Were Mailed but Before Randomization and Receiving Information About Incentives

				Adjusted for age, sex, race, prior screening		
Colorectal cancer test	Mail Only N = 248	Mail and Monetary [a] N = 239	Mail and Lottery [b] N = 259	Monetary vs. Mail Only	Lottery vs. Mail Only	Global
	n (%)	n (%)	n (%)	Difference (95% CI)	Difference (95% CI)	*P* value
Any CRC [c]	167 (67.3)	176 (73.6)	187 (72.2)	7.0 (-0.9, 14.8)	6.2 (-1.4, 13.8)	.08
FIT [d]	154 (62.1)	167 (69.9)	179 (69.1)	8.5 (0.4, 16.6)	8.6 (0.7, 16.6)	.04
Colonoscopy [e]	13 (5.2)	9 (3.8)	8 (3.1)	-1.8 (-5.5, 1.9)	-2.6 (-6.2, 1.1)	.35

Abbreviations: CRC, colorectal cancer; CI, confidence interval; FIT, fecal immunochemical test

[a] Mailed interventions plus $10 cash incentive conditional upon completion of CRC screening

[b] Mailed interventions plus entry into a lottery with a 1 in 10 chance of winning $50 conditional upon completion of CRC screening

[c] Colonoscopy or FIT (no participants had flexible sigmoidoscopies)

[d] FIT completion as the first test (does not include colonoscopy followed by a FIT)

[e] Colonoscopy completion as first test (does not include colonoscopy following a FIT).

© 2019 Green BB et al. *JAMA Network Open.*

eTable2: Subgroup Analyses of Intervention Effects on FIT Completion by Sociodemographic Characteristics With Separate Estimates of the Effects of Mail and Monetary and Mail and Lottery Interventions

	Screened within 6 months			Adjusted differences between groups	
	Mail Only	Mail and Monetary	Mail and Lottery	Monetary vs. Mail Only	Lottery vs. Mail Only
Patient Characteristic	n/total (%)	n/total (%)	n/total (%)	Difference (95% CI)	Difference (95% CI)
Sex					
Male	77/107 (72.0)	68/90 (75.6)	74/95 (77.9)	2.5 (-9.4, 14.5)	6.3 (-5.4, 18.0)
Female	111/177 (62.7)	130/180 (72.2)	130/189 (68.8)	10.4 (1.1, 19.7)	7.6 (-1.6, 16.8)
P value [c]				.37	.99
Age, years					
< 60	100/147 (68.0)	111/147 (75.5)	98/141 (69.5)	7.4 (-1.9, 16.7)	1.6 (-8.2, 11.4)
60 +	88/137 (64.2)	87/123 (70.7)	106/143 (74.1)	6.7 (-4.5, 17.9)	11.3 (0.8, 21.8)
P value [c]				.76	.23
Hispanic					
No	170/254 (66.9)	169/228 (74.1)	178/249 (71.5)	7.8 (-0.02, 15.5)	6.5 (-1.2, 14.1)
Yes	16/28 (57.1)	29/40 (72.5)	25/33 (75.8)	17.0 (-6.5, 40.4)	16.3 (-8.5, 41.1)
P value [c]				.50	.49
Race					
White	96/139 (69.1)	106/146 (72.6)	108/148 (73.0)	4.1 (-6.1, 14.4)	5.8 (-4.3, 15.9)
Black	28/46 (60.9)	31/41 (75.6)	27/37 (73.0)	16.2 (-2.2, 34.7)	13.2 (-6.4, 32.7)
Asian	48/76 (63.2)	42/59 (71.2)	53/73 (72.6)	9.8 (-5.4, 25.1)	11.0 (-3.2, 25.2)
Other	15/22 (68.2)	17/21 (81.0)	13/22 (59.1)	11.3 (-13.1, 35.7)	-9.5 (-36.3, 17.4)
P value [c]				.71	.54
Literacy (needs help interpreting medical documents)					
Never/rarely	166/246 (67.5)	175/233 (75.1)	170/232 (73.3)	8.2 (0.3, 16.0)	7.0 (-0.9, 14.9)
Sometimes/often/always	18/34 (52.9)	19/32 (59.4)	30/44 (68.2)	6.6 (-16.2, 29.4)	14.0 (-6.4, 34.5)
P value [c]				.83	.57
Medicaid					
No	182/270 (67.4)	180/248 (72.6)	185/261 (70.9)	6.3 (-1.3, 13.9)	5.1 (-2.4, 12.5)
Yes	6/14 (42.9)	18/22 (81.8)	19/23 (82.6)	34.2 (4.2, 64.2)	40.4 (11.9, 68.9)
P value [c]				.11	.03
Annual household income					
< $50,000	58/99 (58.6)	66/91 (72.5)	71/102 (69.6)	13.9 (1.5, 26.3)	12.6 (0.3, 24.9)
≥ $50,000	113/164 (68.9)	117/162 (72.2)	117/153 (76.5)	3.7 (-6.1, 13.6)	7.6 (-2.0, 17.2)
P value [c]				.24	.63
Education					
≤ High School	27/41 (65.9)	27/37 (73.0)	40/57 (70.2)	7.5 (-9.7, 24.7)	5.3 (-11.0, 21.5)
Some college	50/90 (55.6)	67/90 (74.4)	64/91 (70.3)	17.7 (4.4, 31.0)	15.7 (2.2, 29.1)
College degree or higher	105/145 (72.4)	98/135 (72.6)	94/126 (74.6)	1.1 (-9.3, 11.6)	2.3 (-8.1, 12.6)
P value [c]				.19	.35
Prior completion of CRC					
No	38/76 (50.0)	41/74 (55.4)	40/78 (51.3)	5.3 (-10.3, 20.9)	2.4 (-12.6, 17.4)
Yes	150/208 (72.1)	157/196 (80.1)	164/206 (79.6)	8.3 (0.3, 16.2)	8.5 (0.6, 16.3)
P value [c]				.52	.32
BMI, kg/m^2					
< 25	52/80 (65.0)	68/85 (80.0)	53/72 (73.6)	15.6 (2.1, 29.1)	10.0 (-4.2, 24.2)
25 to < 35	104/151 (68.9)	101/136 (74.3)	114/158 (72.2)	7.1 (-2.7, 16.9)	4.6 (-5.2, 14.4)
35 or higher	30/47 (63.8)	25/42 (59.5)	34/49 (69.4)	-4.7 (-24.0, 14.6)	9.2 (-8.2, 26.6)

© 2019 Green BB et al. *JAMA Network Open.*

	Screened within 6 months			Adjusted differences between groups	
	Mail Only	Mail and Monetary	Mail and Lottery	Monetary vs. Mail Only	Lottery vs. Mail Only
Patient Characteristic	n/total (%)	n/total (%)	n/total (%)	Difference (95% CI)	Difference (95% CI)
P value [c]				.20	.79
Current tobacco use					
No	174/260 (66.9)	177/241 (73.4)	185/251 (73.7)	7.6 (-0.3, 15.4)	8.6 (1.0, 16.2)
Yes	10/20 (50.0)	14/20 (70.0)	17/28 (60.7)	13.3 (-14.0, 40.6)	3.0 (-24.8, 30.8)
P value [c]				.73	.64
Charlson index score					
0	131/190 (68.9)	129/180 (71.7)	133/178 (74.7)	5.9 (-3.1, 14.8)	8.3 (-0.8, 17.3)
1	30/40 (75.0)	26/32 (81.3)	36/45 (80.0)	3.8 (-14.6, 22.1)	4.5 (-11.5, 20.5)
2 or higher	13/24 (54.2)	23/31 (74.2)	16/31 (51.6)	20.5 (-3.8, 44.7)	4.6 (-20.6, 29.8)
P value [c]				.58	.91

Abbreviations: BMI, Body Mass Index (kg/m^2); CRC, colorectal cancer; CI, confidence interval; FIT, fecal immunochemical test

[a] Mailed interventions plus $10 cash incentive conditional upon completion of CRC screening

[b] Mailed interventions plus entry into a lottery with a 1 in 10 chance of winning $50 conditional upon completion of CRC screening

[c] *P* value for the difference in intervention effect across subgroups, with separate tests/*P* values for the effects of the Mail only; Mail and Monetary; and Mail and Lottery interventions.

Abbreviations: FIT = Fecal Immunochemical Test; CI = confidence interval

© 2019 Green BB et al. *JAMA Network Open.*

eTable 3. Subgroup Analyses of Intervention Effects on FIT Completion by Psychosocial Measures Self-Reported at Baseline With Separate Estimates of the Effects of Mail and Monetary and Mail and Lottery Interventions

	Screened within 6 months			Adjusted differences between groups	
	Mail Only	Mail and Monetary [a]	Mail and Lottery [b]	Monetary vs. Mail Only	Lottery vs. Mail Only
Psychosocial Measures	n/total (%)	n/total (%)	n/total (%)	Difference (95% CI)	Difference (95% CI)
Barriers to CRC screening (mean score, range 1-5)					
1 to < 2	93/137 (67.9)	85/116 (73.3)	88/115 (76.5)	7.6 (-3.4, 18.6)	11.0 (0.0, 21.9)
2 to < 3	62/92 (67.4)	73/96 (76.0)	79/106 (74.5)	7.5 (-5.0, 20.0)	8.0 (-4.0, 20.1)
3 to 5	28/50 (56.0)	36/51 (70.6)	30/51 (58.8)	11.1 (-6.2, 28.5)	2.1 (-15.6, 19.9)
P value[c]				.95	.66
Benefits of CRC screening (mean score, range 1-5)					
1 to < 3	17/31 (54.8)	13/21 (61.9)	16/32 (50.0)	9.2 (-16.9, 35.2)	-4.1 (-28.4, 20.2)
3 to < 4	51/78 (65.4)	69/97 (71.1)	51/71 (71.8)	7.1 (-6.7, 20.8)	9.6 (-4.5, 23.7)
4 to 5	114/169 (67.5)	113/147 (76.9)	130/169 (76.9)	9.2 (-0.5, 18.9)	10.4 (1.1, 19.6)
P value[c]				.96	.47
Self-efficacy for completing CRC screening (mean score, range 1-5)					
1 to < 3	16/33 (48.5)	17/30 (56.7)	14/29 (48.3)	10.8 (-11.4, 32.9)	3.7 (-19.7, 27.0)
3 to < 4	57/80 (71.3)	44/59 (74.6)	59/82 (72.0)	3.8 (-10.5, 18.0)	2.4 (-10.7, 15.5)
4 to 5	109/165 (66.1)	133/174 (76.4)	124/161 (77.0)	10.4 (0.8, 20.0)	11.6 (1.9, 21.3)
P value[c]				.79	.51
General dispositional optimism					
Low optimism (0-13)	35/62 (56.5)	45/57 (78.9)	46/62 (74.2)	23.8 (8.2, 39.4)	17.4 (7.8, 34.1)
Moderate (14-18)	65/107 (60.7)	69/97 (71.1)	77/108 (71.3)	9.7 (-2.4, 21.8)	9.8 (-2.1, 21.6)
High optimism (19-24)	78/103 (75.7)	77/104 (74.0)	70/99 (70.7)	-0.7 (-13.1, 11.7)	0.1 (-11.8, 12.1)
P value[c]				0.06	0.27
defensive information processing: opt-out behavior score (mean of 3 items, range 1-5)					
Lowest tertile (≤ 2)	62/94 (66.0)	66/88 (75.0)	61/84 (72.6)	8.8 (-4.4, 22.0)	6.9 (-6.7, 20.5)
Middle tertile (> 2 to ≤ 3.5)	83/110 (75.5)	84/110 (76.4)	70/92 (76.1)	1.8 (-9.1, 12.8)	1.5 (-10.1, 13.2)
Highest tertile (> 3.5)	38/75 (50.7)	46/67 (68.7)	67/99 (67.7)	18.4 (3.0, 33.7)	18.7 (4.8, 32.6)
P value[c]				0.31	0.24
Single Item from the 14-item Considerations of future consequences scale: “I make decisions or take actions based on how easy they are to do (…like me).					
Not at all/Somewhat not	111/155 (71.6)	99/144 (68.8)	99/138 (71.7)	-4.0 (-14.4, 6.5)	-0.1 (-10.2, 10.1)
Uncertain	24/40 (60.0)	29/38 (76.3)	33/48 (68.8)	23.7 (5.0, 42.4)	13.3 (-6.8, 33.4)
Somewhat/Very much	48/84 (57.1)	62/76 (81.6)	67/90 (74.4)	23.7 (11.1, 36.4)	19.4 (7.0, 31.7)
P value[c]				.001	.06
My risk of colon cancer in the next 10 years, compared to other people my age					
Much lower	58/93 (62.4)	60/84 (71.4)	74/100 (74.0)	10.1 (-3.0, 23.2)	14.3 (2.1, 26.4)
A little lower	53/75 (70.7)	59/76 (77.6)	50/67 (74.6)	8.4 (-5.5, 22.2)	7.9 (-6.1, 21.9)
Average	60/90 (66.7)	66/89 (74.2)	64/91 (70.3)	5.6 (-7.4, 18.6)	2.7 (-10.3, 15.6)
A little/much higher	10/17 (58.8)	7/12 (58.3)	7/14 (50.0)	1.0 (-33.8, 35.8)	-12.1 (-47.6, 23.3)
P value[c]				.94	.39

Abbreviations: CRC, colorectal cancer; CI, confidence interval

[a] +Monetary = Mailed interventions plus $10 cash incentive conditional upon completion of colorectal cancer screening

© 2019 Green BB et al. *JAMA Network Open.*

[b] +Lottery = Mailed interventions plus entry into a lottery with a 1 in 10 chance of winning $50 conditional upon completion of colorectal cancer screening
[c] *P* value for the difference in intervention effect across subgroups, with separate tests/*P* values for the effects of the +Monetary and +Lottery interventions.

© 2019 Green BB et al. *JAMA Network Open.*
