# Supplementary material for: Financial Incentives to Increase Colorectal Cancer Screening Uptake and Decrease Disparities: A Randomized Clinical Trial
Source: JAMA Netw Open. Author manuscript; Available in PMC 2019 Oct 12. (PMC6789432; doi:10.1001/jamanetworkopen.2019.6570)
Supplement: Supplement 3 — Data Sharing Statement [file NIHMS1047899-supplement-Supplement_3.pdf]

Data Sharing Statement

Green. Financial Incentives to Increase Colorectal Cancer Screening Uptake and Decrease Disparities. *JAMA Netw Open*. Published July 05, 2019. 10.1001/jamanetworkopen.2019.6570

Data
Data available: Yes
Data types: Deidentified participant data, Data dictionary
How to access data: By request to Bev.B.Green@kp.org
When available: With publication

Supporting Documents
Document types: None

Additional Information
Who can access the data: Researchers whose proposed use of the data has been approved
Types of analyses: De-identified participant data, Data dictionary
Mechanisms of data availability: Without investigator support
Any additional restrictions: Data cannot be used to attempt to re-identify any sites or patients and contact them in any manner.
