# Supplementary material for: Financial Incentives to Increase Colorectal Cancer Screening Uptake and Decrease Disparities: A Randomized Clinical Trial
Source: JAMA Netw Open. Author manuscript; Available in PMC 2019 Oct 12. (PMC6789432; doi:10.1001/jamanetworkopen.2019.6570)
Supplement: Supplementary Online Content [file NIHMS1047899-supplement-Supplementary_Online_Content.docx]

**Supplementary Online Content**

Green BB, Anderson ML, Cook AJ, et al. Financial incentives to increase colorectal cancer screening uptake and decrease disparities: a randomized clinical trial. *JAMA Netw Open*. 2010;2(7):e196570. doi:10.1001/jamanetworkopen.2019.6570

**eTable 1.** Proportions Tested for Colorectal Cancer Within 6 Months From Sensitivity Analysis Excluding Participants Who Tested After Questionnaires Were Mailed but Before Randomization and Receiving Information About Incentives

**eTable 2.** Subgroup Analyses of Intervention Effects on FIT Completion by Sociodemographic Characteristics With Separate Estimates of the Effects of Mail and Monetary and Mail and Lottery Interventions

**eTable 3.** Subgroup Analyses of Intervention Effects on FIT Completion by Psychosocial Measures Self-Reported at Baseline With Separate Estimates of the Effects of Mail and Monetary and Mail and Lottery Interventions

**This supplementary material has been provided by the authors to give readers additional information about their work.**

**eTable 1.** Proportions Tested for Colorectal Cancer Within 6 Months From Sensitivity Analysis Excluding Participants Who Tested After Questionnaires Were Mailed but Before Randomization and Receiving Information About Incentives

|  |  |  |  | Adjusted for age, sex, race, prior screening | | |
| --- | --- | --- | --- | --- | --- | --- |
| Colorectal cancer test | Mail Only  N = 248 | Mail and Monetary ^a^  N = 239 | Mail and Lottery ^b^  N = 259 | Monetary vs.  Mail Only | Lottery vs.  Mail Only | Global |
|  | n (%) | n (%) | n (%) | Difference (95% CI) | Difference  (95% CI) | *P* value |
| Any CRC ^c^ | 167 (67.3) | 176 (73.6) | 187 (72.2) | 7.0 (-0.9, 14.8) | 6.2 (-1.4, 13.8) | .08 |
| FIT ^d^ | 154 (62.1) | 167 (69.9) | 179 (69.1) | 8.5 (0.4, 16.6) | 8.6 (0.7, 16.6) | .04 |
| Colonoscopy ^e^ | 13 (5.2) | 9 (3.8) | 8 (3.1) | -1.8 (-5.5, 1.9) | -2.6 (-6.2, 1.1) | .35 |

Abbreviations: CRC, colorectal cancer; CI, confidence interval; FIT, fecal immunochemical test

^a^ Mailed interventions plus $10 cash incentive conditional upon completion of CRC screening

^b^ Mailed interventions plus entry into a lottery with a 1 in 10 chance of winning $50 conditional upon completion of CRC screening

^c^ Colonoscopy or FIT (no participants had flexible sigmoidoscopies)

^d^ FIT completion as the first test (does not include colonoscopy followed by a FIT)

^e^ Colonoscopy completion as first test (does not include colonoscopy following a FIT).

**eTable2: Subgroup Analyses of Intervention Effects on FIT Completion by Sociodemographic Characteristics With Separate Estimates of the Effects of Mail and Monetary and Mail and Lottery Interventions**

|  | Screened within 6 months | | | | Adjusted differences between groups | | |
| --- | --- | --- | --- | --- | --- | --- | --- |
|  | Mail Only | Mail and Monetary | Mail and  Lottery | Monetary vs.  Mail Only | | Lottery vs.  Mail Only |  |
| Patient Characteristic | n/total (%) | n/total (%) | n/total (%) | Difference  (95% CI) | | Difference  (95% CI) |  |
| Sex | | | | | | | |
| Male | 77/107 (72.0) | 68/90 (75.6) | 74/95 (77.9) | 2.5 (-9.4, 14.5) | | 6.3 (-5.4, 18.0) |  |
| Female | 111/177 (62.7) | 130/180 (72.2) | 130/189 (68.8) | 10.4 (1.1, 19.7) | | 7.6 (-1.6, 16.8) |  |
| *P* value ^c^ |  |  |  | .37 | | .99 |  |
| Age, years | | | | | | | |
| < 60 | 100/147 (68.0) | 111/147 (75.5) | 98/141 (69.5) | 7.4 (-1.9, 16.7) | | 1.6 (-8.2, 11.4) |  |
| 60 + | 88/137 (64.2) | 87/123 (70.7) | 106/143 (74.1) | 6.7 (-4.5, 17.9) | | 11.3 (0.8, 21.8) |  |
| *P* value ^c^ |  |  |  | .76 | | .23 |  |
| Hispanic | | | | | | | |
| No | 170/254 (66.9) | 169/228 (74.1) | 178/249 (71.5) | 7.8 (-0.02, 15.5) | | 6.5 (-1.2, 14.1) |  |
| Yes | 16/28 (57.1) | 29/40 (72.5) | 25/33 (75.8) | 17.0 (-6.5, 40.4) | | 16.3 (-8.5, 41.1) |  |
| *P* value ^c^ |  |  |  | .50 | | .49 |  |
| Race | | | | | | | |
| White | 96/139 (69.1) | 106/146 (72.6) | 108/148 (73.0) | 4.1 (-6.1, 14.4) | | 5.8 (-4.3, 15.9) |  |
| Black | 28/46 (60.9) | 31/41 (75.6) | 27/37 (73.0) | 16.2 (-2.2, 34.7) | | 13.2 (-6.4, 32.7) |  |
| Asian | 48/76 (63.2) | 42/59 (71.2) | 53/73 (72.6) | 9.8 (-5.4, 25.1) | | 11.0 (-3.2, 25.2) |  |
| Other | 15/22 (68.2) | 17/21 (81.0) | 13/22 (59.1) | 11.3 (-13.1, 35.7) | | -9.5 (-36.3, 17.4) |  |
| *P* value ^c^ |  |  |  | .71 | | .54 |  |
| Literacy (needs help interpreting medical documents) | | | | | | | |
| Never/rarely | 166/246 (67.5) | 175/233 (75.1) | 170/232 (73.3) | 8.2 (0.3, 16.0) | | 7.0 (-0.9, 14.9) |  |
| Sometimes/often/always | 18/34 (52.9) | 19/32 (59.4) | 30/44 (68.2) | 6.6 (-16.2, 29.4) | | 14.0 (-6.4, 34.5) |  |
| *P* value ^c^ |  |  |  | .83 | | .57 |  |
| Medicaid | | | | | | | |
| No | 182/270 (67.4) | 180/248 (72.6) | 185/261 (70.9) | 6.3 (-1.3, 13.9) | | 5.1 (-2.4, 12.5) |  |
| Yes | 6/14 (42.9) | 18/22 (81.8) | 19/23 (82.6) | 34.2 (4.2, 64.2) | | 40.4 (11.9, 68.9) |  |
| *P* value ^c^ |  |  |  | .11 | | .03 |  |
| Annual household income | | | | | | | |
| < $50,000 | 58/99 (58.6) | 66/91 (72.5) | 71/102 (69.6) | 13.9 (1.5, 26.3) | | 12.6 (0.3, 24.9) |  |
| ≥ $50,000 | 113/164 (68.9) | 117/162 (72.2) | 117/153 (76.5) | 3.7 (-6.1, 13.6) | | 7.6 (-2.0, 17.2) |  |
| *P* value ^c^ |  |  |  | .24 | | .63 |  |
| Education | | | | | | | |
| ≤ High School | 27/41 (65.9) | 27/37 (73.0) | 40/57 (70.2) | 7.5 (-9.7, 24.7) | | 5.3 (-11.0, 21.5) |  |
| Some college | 50/90 (55.6) | 67/90 (74.4) | 64/91 (70.3) | 17.7 (4.4, 31.0) | | 15.7 (2.2, 29.1) |  |
| College degree or higher | 105/145 (72.4) | 98/135 (72.6) | 94/126 (74.6) | 1.1 (-9.3, 11.6) | | 2.3 (-8.1, 12.6) |  |
| *P* value ^c^ |  |  |  | .19 | | .35 |  |
| Prior completion of CRC | | | | | | | |
| No | 38/76 (50.0) | 41/74 (55.4) | 40/78 (51.3) | 5.3 (-10.3, 20.9) | | 2.4 (-12.6, 17.4) |  |
| Yes | 150/208 (72.1) | 157/196 (80.1) | 164/206 (79.6) | 8.3 (0.3, 16.2) | | 8.5 (0.6, 16.3) |  |
| *P* value ^c^ |  |  |  | .52 | | .32 |  |
| BMI, kg/m^2^ | | | | | | | |
| < 25 | 52/80 (65.0) | 68/85 (80.0) | 53/72 (73.6) | 15.6 (2.1, 29.1) | | 10.0 (-4.2, 24.2) |  |
| 25 to < 35 | 104/151 (68.9) | 101/136 (74.3) | 114/158 (72.2) | 7.1 (-2.7, 16.9) | | 4.6 (-5.2, 14.4) |  |
| 35 or higher | 30/47 (63.8) | 25/42 (59.5) | 34/49 (69.4) | -4.7 (-24.0, 14.6) | | 9.2 (-8.2, 26.6) |  |
| *P* value ^c^ |  |  |  | .20 | | .79 |  |
| Current tobacco use | | | | | | | |
| No | 174/260 (66.9) | 177/241 (73.4) | 185/251 (73.7) | 7.6 (-0.3, 15.4) | | 8.6 (1.0, 16.2) |  |
| Yes | 10/20 (50.0) | 14/20 (70.0) | 17/28 (60.7) | 13.3 (-14.0, 40.6) | | 3.0 (-24.8, 30.8) |  |
| *P* value ^c^ |  |  |  | .73 | | .64 |  |
| Charlson index score | | | | | | | |
| 0 | 131/190 (68.9) | 129/180 (71.7) | 133/178 (74.7) | 5.9 (-3.1, 14.8) | | 8.3 (-0.8, 17.3) |  |
| 1 | 30/40 (75.0) | 26/32 (81.3) | 36/45 (80.0) | 3.8 (-14.6, 22.1) | | 4.5 (-11.5, 20.5) |  |
| 2 or higher | 13/24 (54.2) | 23/31 (74.2) | 16/31 (51.6) | 20.5 (-3.8, 44.7) | | 4.6 (-20.6, 29.8) |  |
| *P* value ^c^ |  |  |  | .58 | | .91 |  |

Abbreviations: BMI, Body Mass Index (kg/m^2^); CRC, colorectal cancer; CI, confidence interval; FIT, fecal immunochemical test

^a^ Mailed interventions plus $10 cash incentive conditional upon completion of CRC screening

^b^ Mailed interventions plus entry into a lottery with a 1 in 10 chance of winning $50 conditional upon completion of CRC screening

*^c^ P* value for the difference in intervention effect across subgroups, with separate tests/*P* values for the effects of the Mail only; Mail and Monetary; and Mail and Lottery interventions.

Abbreviations: FIT = Fecal Immunochemical Test; CI = confidence interval

**eTable 3. Subgroup Analyses of Intervention Effects on FIT Completion by Psychosocial Measures Self-Reported at Baseline With Separate Estimates of the Effects of Mail and Monetary and Mail and Lottery Interventions**

|  | Screened within 6 months | | | Adjusted differences between groups | |
| --- | --- | --- | --- | --- | --- |
|  | Mail Only | Mail and Monetary ^a^ | Mail and Lottery ^b^ | Monetary vs.  Mail Only | Lottery vs.  Mail Only |
| Psychosocial Measures | n/total (%) | n/total (%) | n/total (%) | Difference (95% CI) | Difference (95% CI) |
| Barriers to CRC screening (mean score, range 1-5) | | | | | |
| 1 to < 2 | 93/137 (67.9) | 85/116 (73.3) | 88/115 (76.5) | 7.6 (-3.4, 18.6) | 11.0 (0.0, 21.9) |
| 2 to < 3 | 62/92 (67.4) | 73/96 (76.0) | 79/106 (74.5) | 7.5 (-5.0, 20.0) | 8.0 (-4.0, 20.1) |
| 3 to 5 | 28/50 (56.0) | 36/51 (70.6) | 30/51 (58.8) | 11.1 (-6.2, 28.5) | 2.1 (-15.6, 19.9) |
| *P* value^c^ |  |  |  | .95 | .66 |
| Benefits of CRC screening (mean score, range 1-5) | | | | | |
| 1 to < 3 | 17/31 (54.8) | 13/21 (61.9) | 16/32 (50.0) | 9.2 (-16.9, 35.2) | -4.1 (-28.4, 20.2) |
| 3 to < 4 | 51/78 (65.4) | 69/97 (71.1) | 51/71 (71.8) | 7.1 (-6.7, 20.8) | 9.6 (-4.5, 23.7) |
| 4 to 5 | 114/169 (67.5) | 113/147 (76.9) | 130/169 (76.9) | 9.2 (-0.5, 18.9) | 10.4 (1.1, 19.6) |
| *P* value^c^ |  |  |  | .96 | .47 |
| Self-efficacy for completing CRC screening (mean score, range 1-5) | | | | | |
| 1 to < 3 | 16/33 (48.5) | 17/30 (56.7) | 14/29 (48.3) | 10.8 (-11.4, 32.9) | 3.7 (-19.7, 27.0) |
| 3 to < 4 | 57/80 (71.3) | 44/59 (74.6) | 59/82 (72.0) | 3.8 (-10.5, 18.0) | 2.4 (-10.7, 15.5) |
| 4 to 5 | 109/165 (66.1) | 133/174 (76.4) | 124/161 (77.0) | 10.4 (0.8, 20.0) | 11.6 (1.9, 21.3) |
| *P* value^c^ |  |  |  | .79 | .51 |
| General dispositional optimism | | | | | |
| Low optimism (0-13) | 35/62 (56.5) | 45/57 (78.9) | 46/62 (74.2) | 23.8 (8.2, 39.4) | 17.4 (7.8, 34.1) |
| Moderate (14-18) | 65/107 (60.7) | 69/97 (71.1) | 77/108 (71.3) | 9.7 (-2.4, 21.8) | 9.8 (-2.1, 21.6) |
| High optimism (19-24) | 78/103 (75.7) | 77/104 (74.0) | 70/99 (70.7) | -0.7 (-13.1, 11.7) | 0.1 (-11.8, 12.1) |
| *P* value^c^ |  |  |  | 0.06 | 0.27 |
| defensive information processing: opt-out behavior score (mean of 3 items, range 1-5) | | | | | |
| Lowest tertile (≤ 2) | 62/94 (66.0) | 66/88 (75.0) | 61/84 (72.6) | 8.8 (-4.4, 22.0) | 6.9 (-6.7, 20.5) |
| Middle tertile (> 2 to ≤ 3.5) | 83/110 (75.5) | 84/110 (76.4) | 70/92 (76.1) | 1.8 (-9.1, 12.8) | 1.5 (-10.1, 13.2) |
| Highest tertile (> 3.5) | 38/75 (50.7) | 46/67 (68.7) | 67/99 (67.7) | 18.4 (3.0, 33.7) | 18.7 (4.8, 32.6) |
| *P* value^c^ |  |  |  | 0.31 | 0.24 |
| Single Item from the 14-item Considerations of future consequences scale: “I make decisions or take actions based on how easy they are to do (…like me). | | | | | |
| Not at all/Somewhat not | 111/155 (71.6) | 99/144 (68.8) | 99/138 (71.7) | -4.0 (-14.4, 6.5) | -0.1 (-10.2, 10.1) |
| Uncertain | 24/40 (60.0) | 29/38 (76.3) | 33/48 (68.8) | 23.7 (5.0, 42.4) | 13.3 (-6.8, 33.4) |
| Somewhat/Very much | 48/84 (57.1) | 62/76 (81.6) | 67/90 (74.4) | 23.7 (11.1, 36.4) | 19.4 (7.0, 31.7) |
| *P* value^c^ |  |  |  | .001 | .06 |
| My risk of colon cancer in the next 10 years, compared to other people my age | | | | | |
| Much lower | 58/93 (62.4) | 60/84 (71.4) | 74/100 (74.0) | 10.1 (-3.0, 23.2) | 14.3 (2.1, 26.4) |
| A little lower | 53/75 (70.7) | 59/76 (77.6) | 50/67 (74.6) | 8.4 (-5.5, 22.2) | 7.9 (-6.1, 21.9) |
| Average | 60/90 (66.7) | 66/89 (74.2) | 64/91 (70.3) | 5.6 (-7.4, 18.6) | 2.7 (-10.3, 15.6) |
| A little/much higher | 10/17 (58.8) | 7/12 (58.3) | 7/14 (50.0) | 1.0 (-33.8, 35.8) | -12.1 (-47.6, 23.3) |
| *P* value^c^ |  |  |  | .94 | .39 |

Abbreviations: CRC, colorectal cancer; CI, confidence interval

^a^ +Monetary = Mailed interventions plus $10 cash incentive conditional upon completion of colorectal cancer screening

^b^ +Lottery = Mailed interventions plus entry into a lottery with a 1 in 10 chance of winning $50 conditional upon completion of colorectal cancer screening

*^c^* *P* value for the difference in intervention effect across subgroups, with separate tests/*P* values for the effects of the +Monetary and +Lottery interventions.
